# Supplementary figures and images for: Compressive instabilities enable cell-induced extreme densification patterns in the fibrous extracellular matrix: Discrete model predictions
Source: PLoS Comput Biol. 2024 Jul 1;20(7):e1012238. doi: 10.1371/journal.pcbi.1012238 (PMC11244807; doi:10.1371/journal.pcbi.1012238)

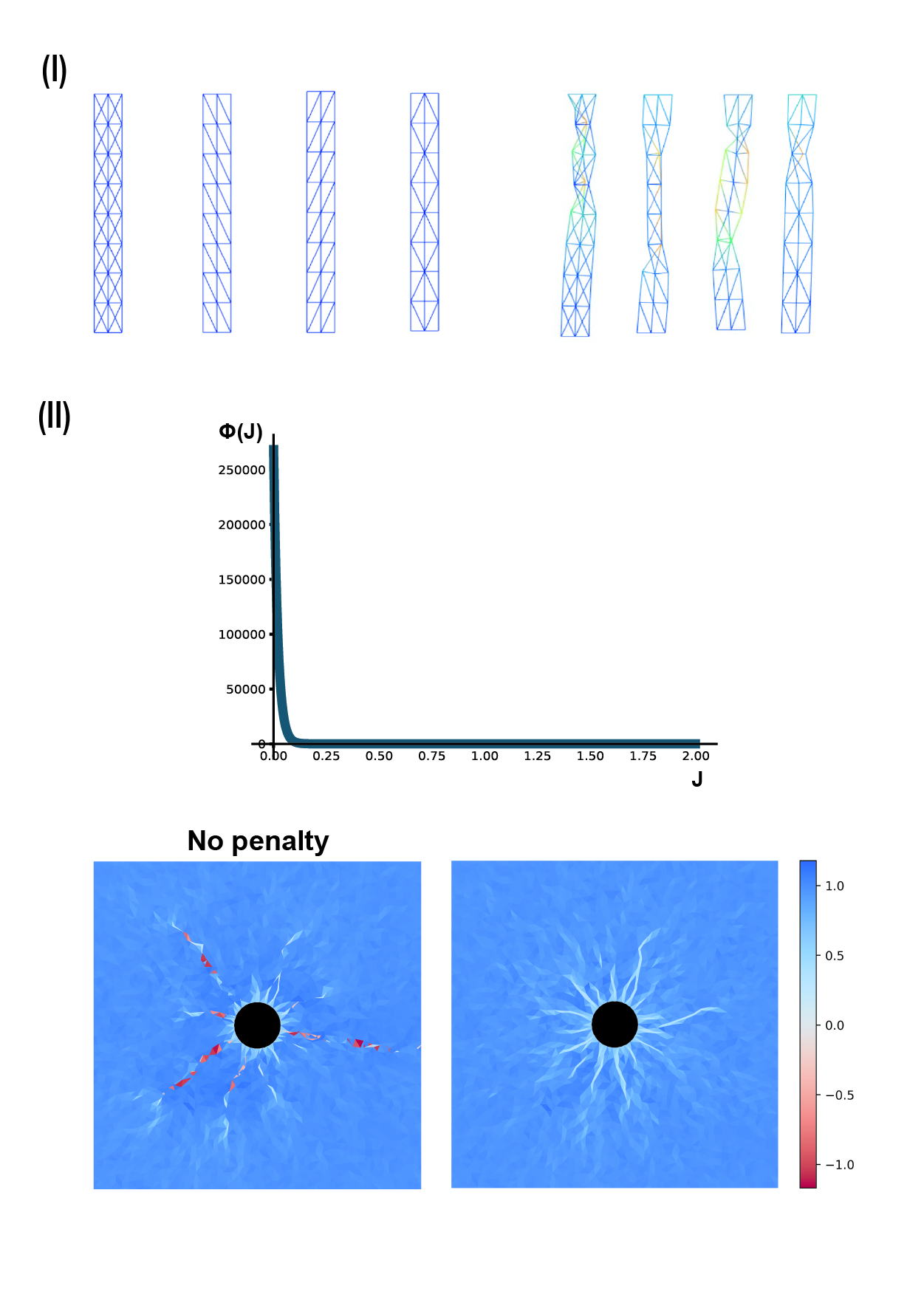

Supplement: S1 Fig — (I) Various presentations of triangulated rectangular truss elements. Each edge in the structures represents a linear spring. Dirichlet boundary conditions were applied on the upper boundary nodes by imposing a displacement u = (h, 0.0), h being the scale to x direction. Deformed structures contain triangles that have changed orientation, resulting in interpenetration of matter. (II) Top: Penalty term Φ(J) = exp(−Q(J − b)), where J is ratio of deformed to undeformed oriented triangle area. Q > 0 is large and b > 0 is small constant. As a result, negative values of J have high energy cost, whereas positive values have negligible contribution to the network’s total energy. Bottom: Simulations of a cell contracting by 50%, either with or without the penalty term for the area ratio J. Without penalizing J, the optimizer finds solutions that are physically unacceptable, as J < 0 corresponds to elements (red) that changed orientation. Colorbar: J values. (TIF) [file pcbi.1012238.s001.tif]

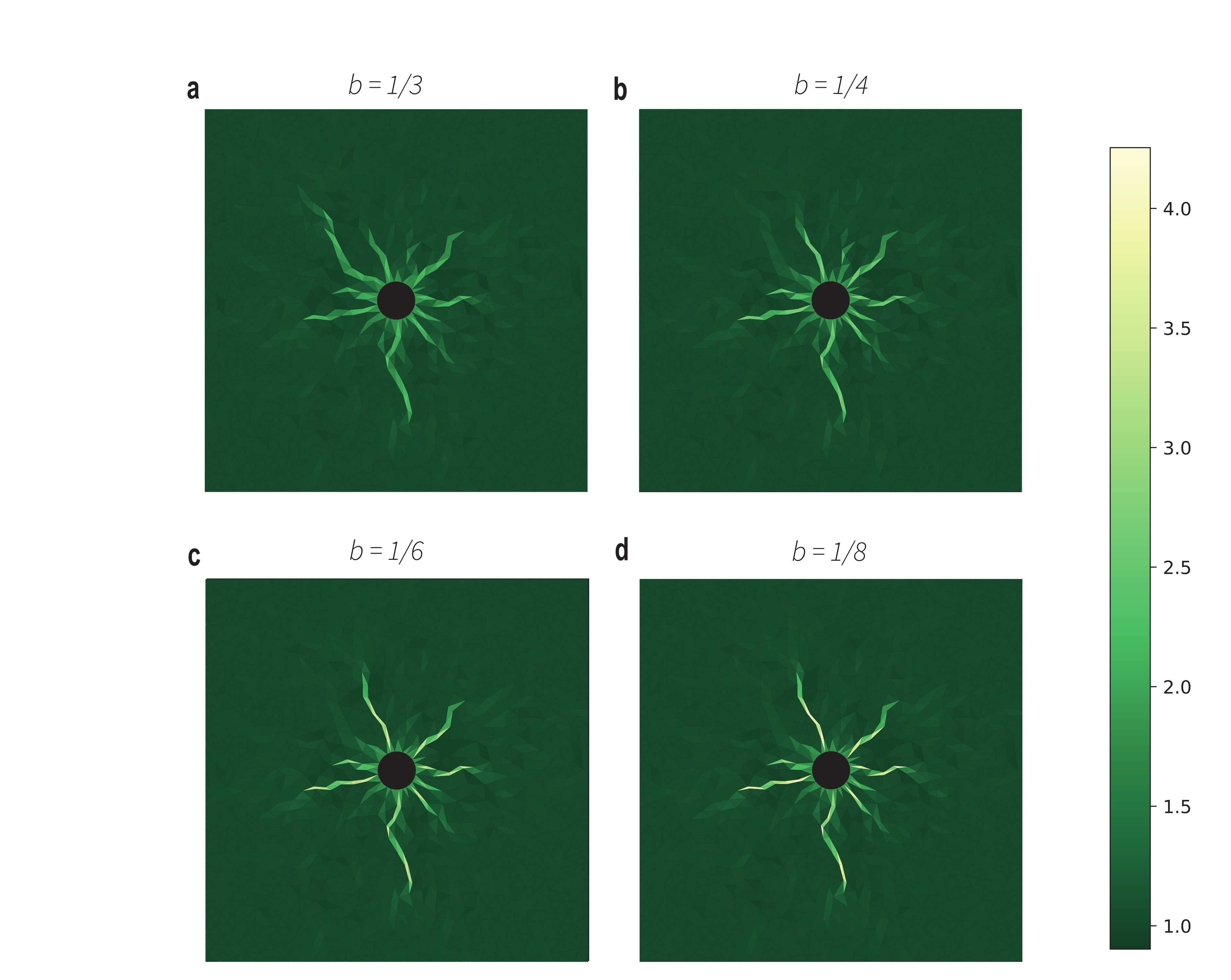

Supplement: S2 Fig — The penalty term is given by Φ(J) = exp(−Q(J − b)), where J is ratio of deformed to undeformed oriented triangle area. We illustrate simulations of a cell contracting by 50%, employing the penalty term with Q = 50 and b = 1/3, 1/4, 1/6 or 1/8. The obtained solutions exhibit qualitative similarity. Similar results are observed when varying Q while keeping b constant. Colorbar: densification ratio of the deformed networks. (TIF) [file pcbi.1012238.s002.tif]

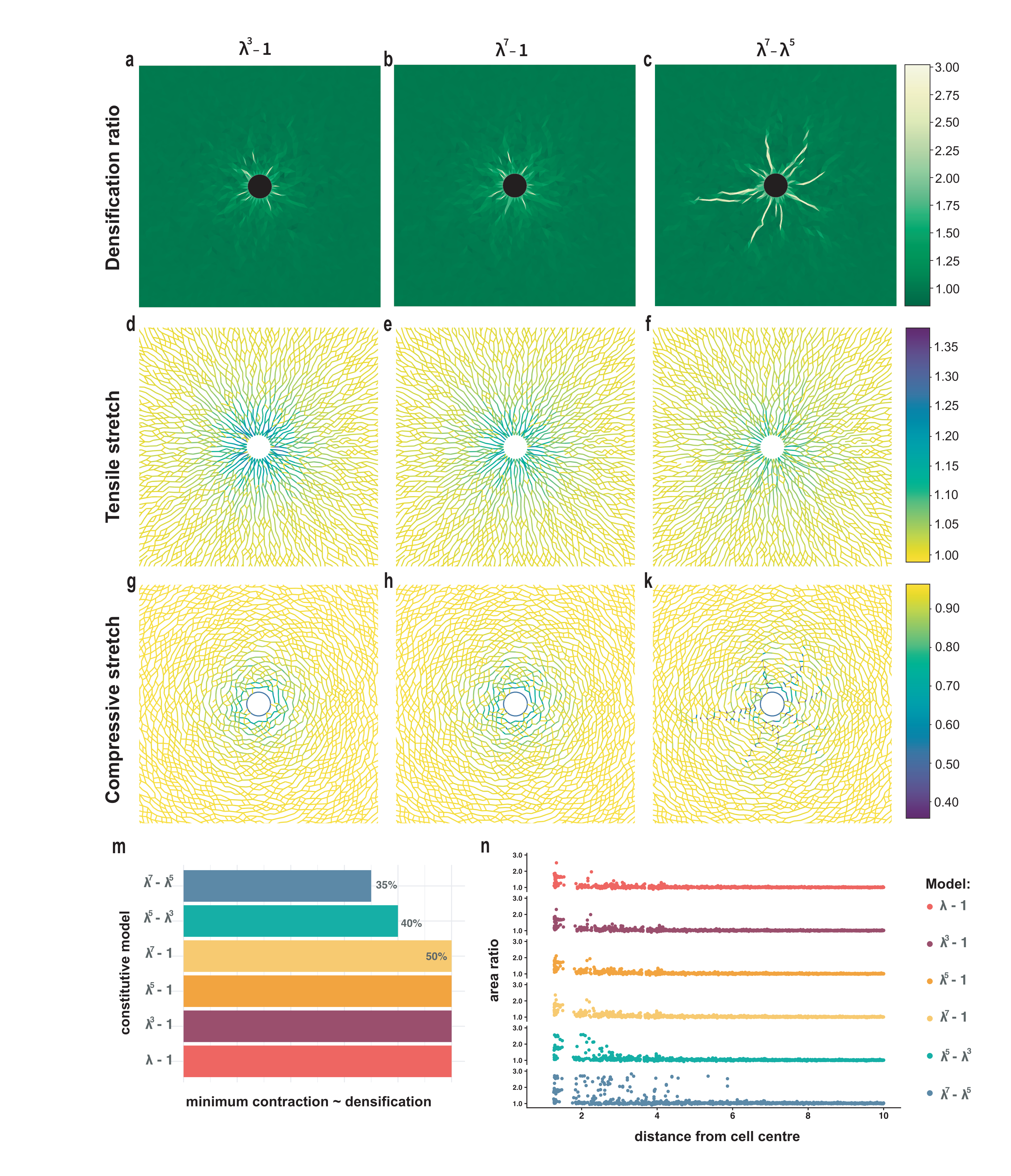

Supplement: S3 Fig — Complementary to Results, Fig 3 containing simulations of a single cell at 50% contraction with Family-1 models λ3 − 1 and λ7 − 1 and Family-2 model λ7 − λ5. (a-c) Densification ratio of triangular elements (color plot) in deformed networks (d-f) tensile stretches and (g-k) compressive stretches in deformed fibers. (m) Minimum contraction required for densification to be evident for each one of the models studied. (n) Simulations with various models of one cell contracting at 50%; x axis: triangular element distance from cell center, y axis: element densification ratio. Colorbars: (a-c) densification ratio ϱ of the deformed networks, (d-k) fiber stretch λ. (TIFF) [file pcbi.1012238.s003.tiff]

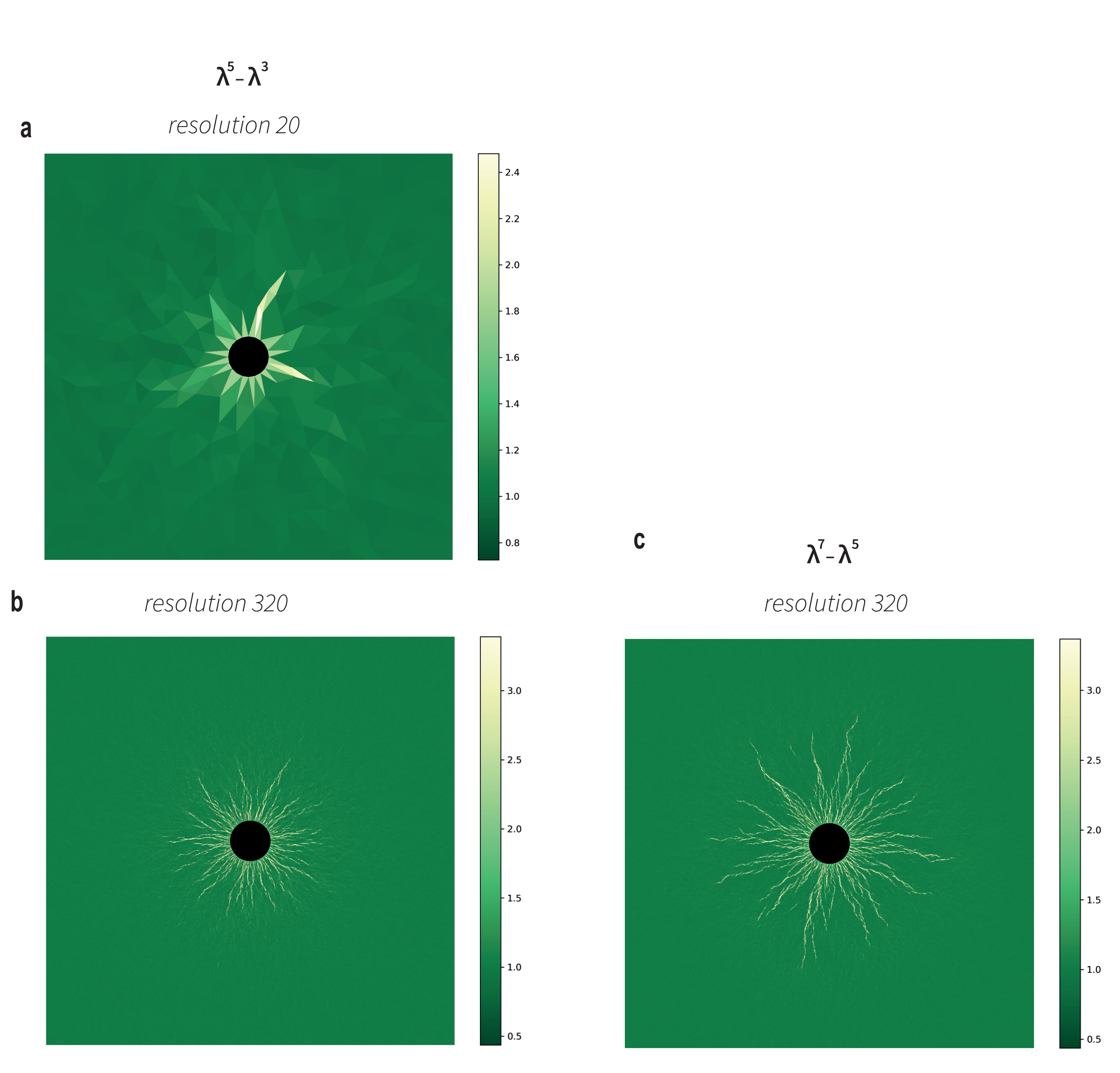

Supplement: S4 Fig — (a) A cell or small particle whose radius is a few fiber lengths (fiber network is visible as the mesh) produces very few densified bands with densification ratio above 2, whereas at the same contraction level of 50% a larger particle produces a great number of radial hairs with higher densification ratio exceeding 3. The model in a) and b) is Family 2 S(λ) = λ5 − λ3. (c) Same mesh (fiber network) as in (b) but with the Family 2 S(λ) = λ7 − λ5 model. Hairs are substantially longer than in (b). Colorbar: densification ratio of the deformed network. (TIFF) [file pcbi.1012238.s004.tiff]

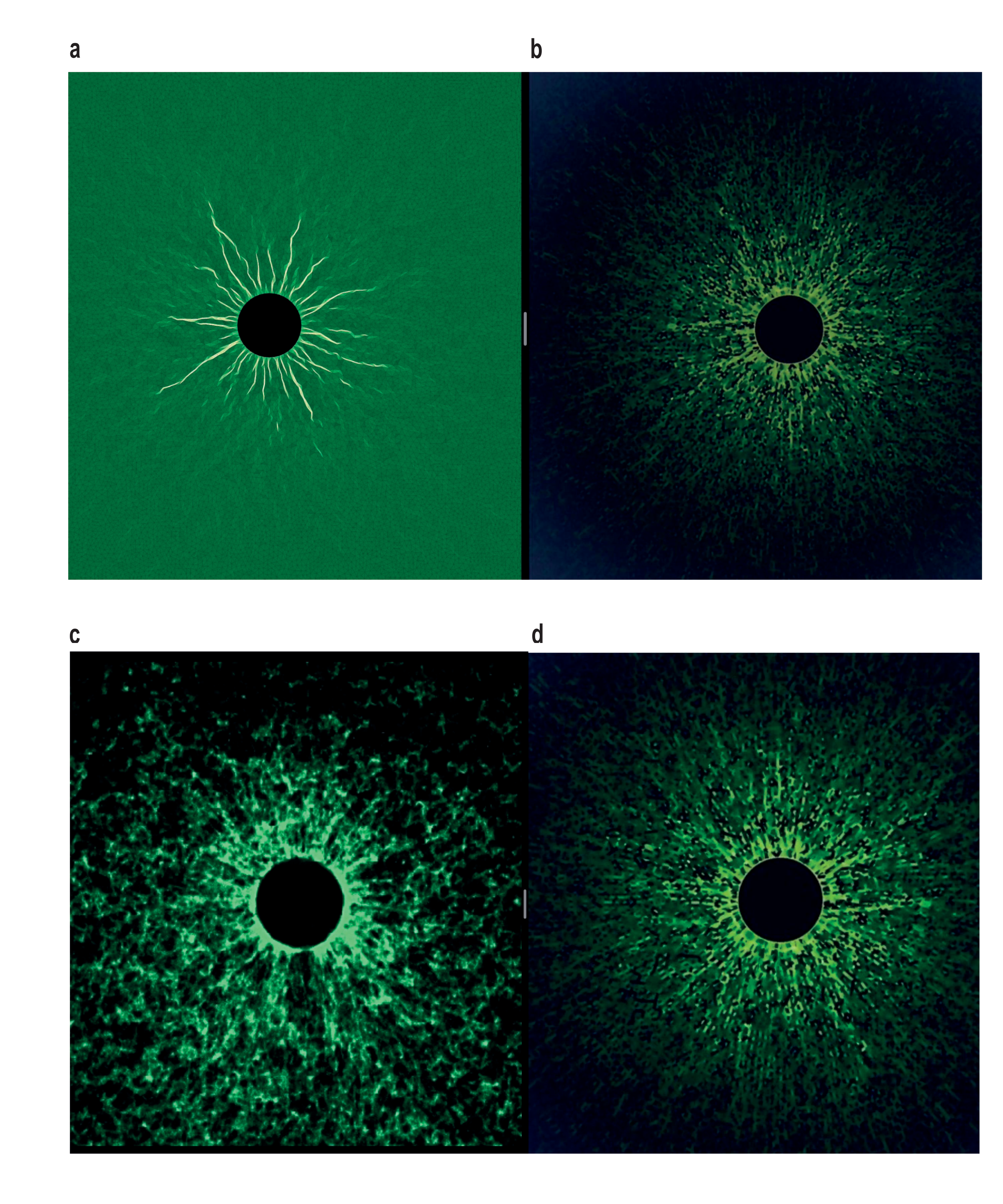

Supplement: S5 Fig — Simulated area densification (a) and fiber density (b), both obtained from the same simulation of an active particle undergoing 50% contraction. Area densification ratio is much more inhomogeneous than fiber density, and densified tracts appear fewer and further apart in (a) versus (b). The length of densified tracts as defined by area densification (a) is much more objective than when defined by fiber density (b), because the former exhibits a sharp discontinuity and is largely independent of photographic scales such as exposure, contrast, brightness etc. Also it is more difficult to compare it to available experimental images, such as (c) (adapted with uniform contrast increase from Fig 5b of [19] with permission) which are sensitive to photography and its electronic reproduction at various stages. Here (c) is juxtaposed with a simulation (d) for comparison. Unfortunately, the light intensity versus fiber density curve from experiments reported in [19] is not available, and there is a subjective choice of brightness/contrast scale in both experimental images (c) and simulations (b), (d). However, it is clear that there are radial hair structures in both. Their apparent length depends on brightness vs fiber density scales that are to some extent arbitrary, but the topology and pattern is similar and much more objective. In (c) we have increased the experimental image contrast uniformly across the image, to emphasise the nonuniform fibers emanating from the particle. (TIFF) [file pcbi.1012238.s005.tiff]

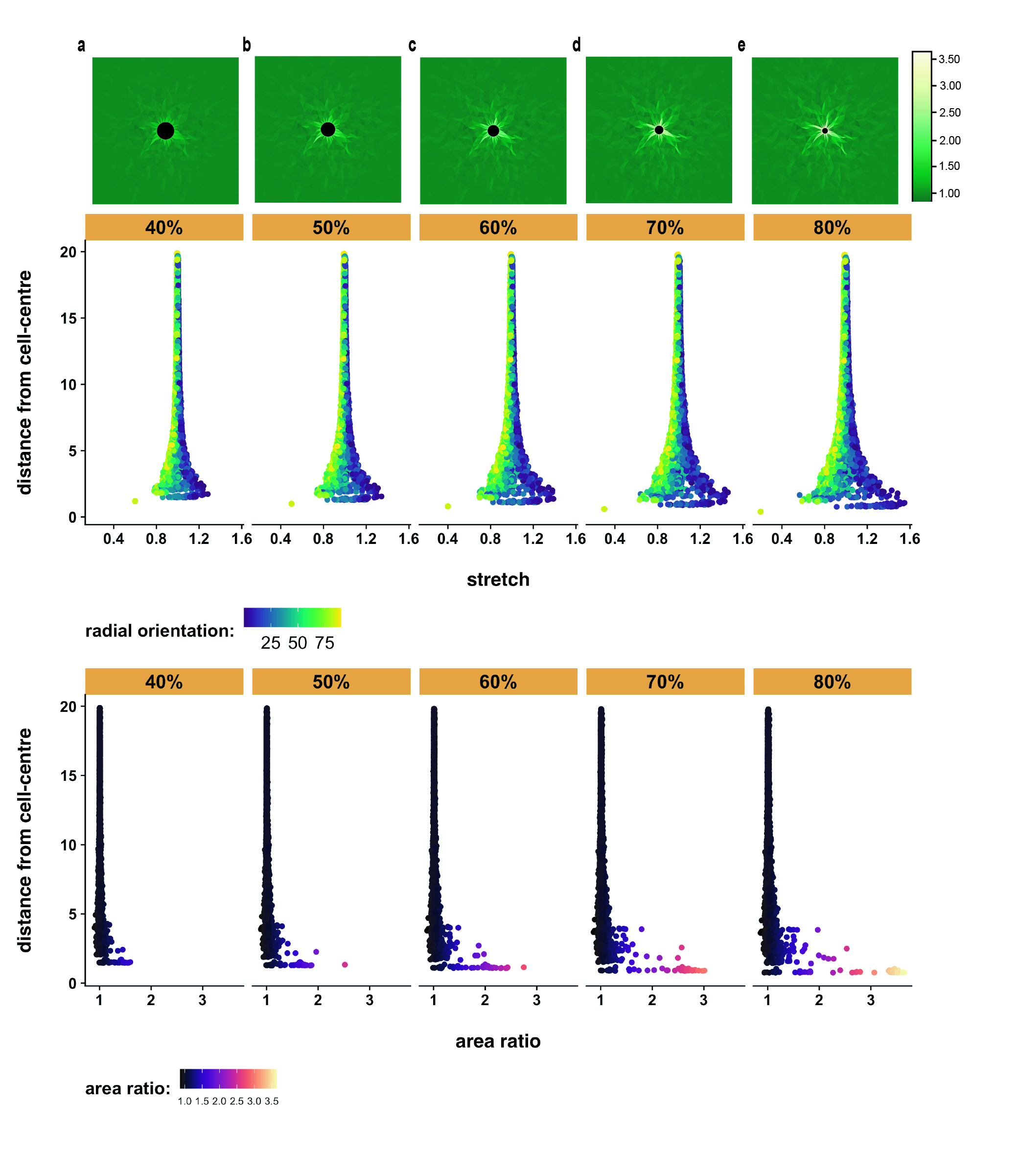

Supplement: S6 Fig — Complementary to Results, Fig 4. Simulations with the linear Family-1 model S(λ) = λ − 1 of a cell contracting in the range 5%–80%. Top: densification ratio ϱ color plot at each indicated contraction step. Middle: tree diagrams, fiber distance from cell center versus fiber stretch for all fibers in the network at each contraction step, x axis: fiber stretch, y axis: fiber distance from cell center. Bottom: triangular element distance from cell center versus densification area ratio, x axis: densification area ratio ϱ, y axis: triangular element distance from cell center. (TIF) [file pcbi.1012238.s006.tif]

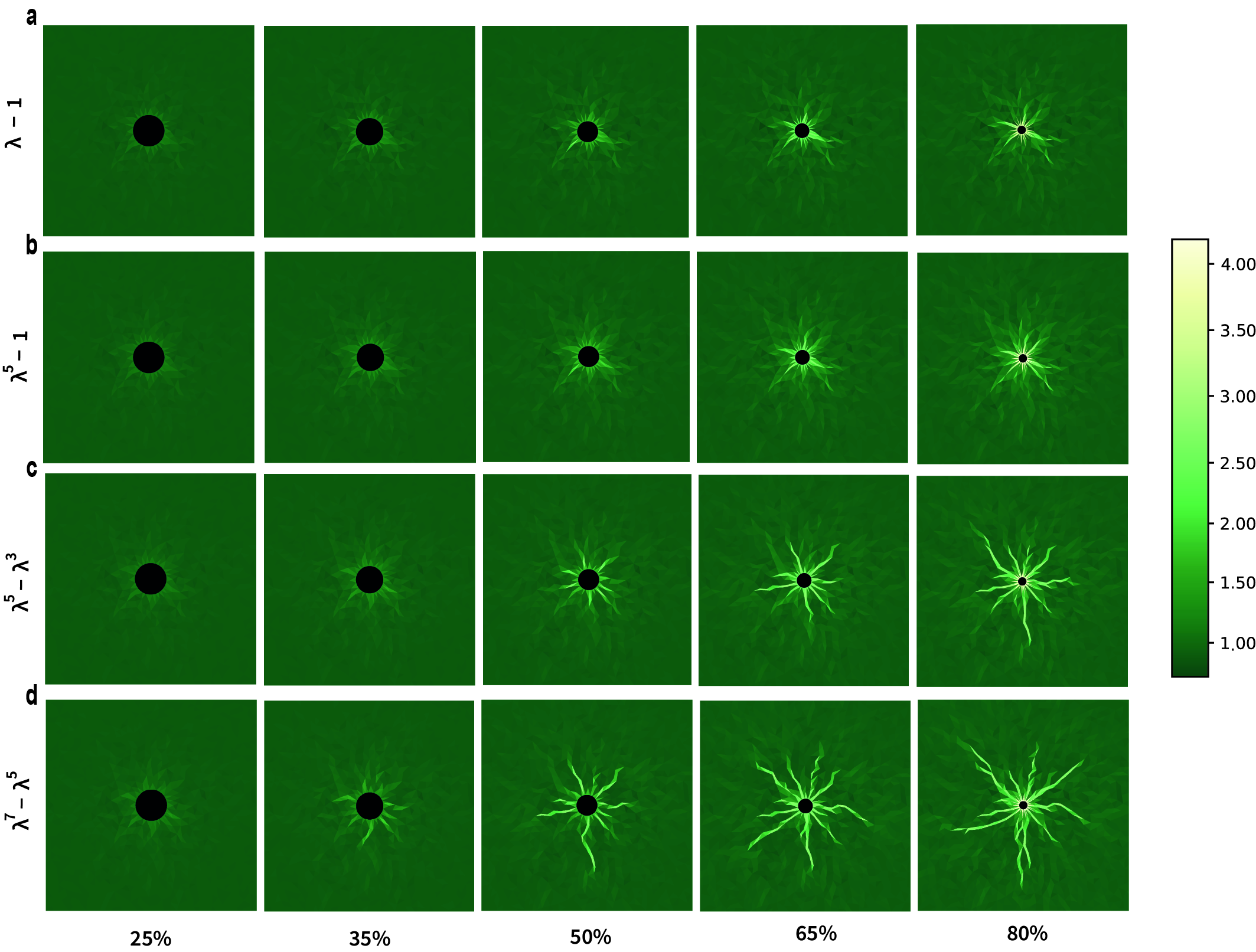

Supplement: S7 Fig — (a-b) As contraction level rises, densification strengthens in the close proximity of the cell for Family-1 models. The bands consisting of densified elements do not propagate far from the cell boundary, reaching as far as 3 deformed cell radii at 80%. On the contrary, in Family-2 simulations (c-d) densification is evident at much lower contraction levels, 35%. With increased contraction, more densified bands are generated and extend substantially further into the matrix. Colorbar: densification ratio ϱ of deformed networks. (TIF) [file pcbi.1012238.s007.tif]

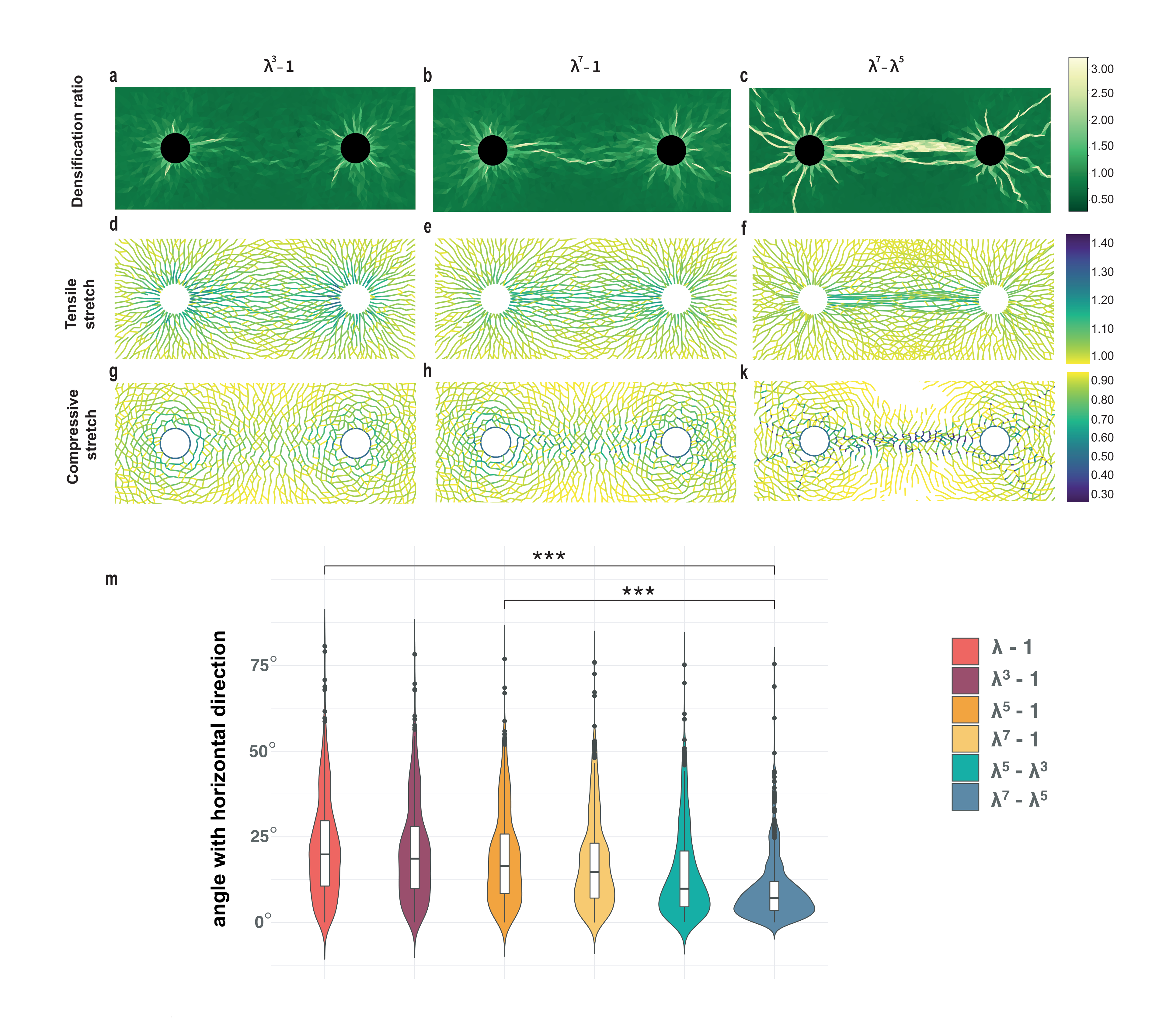

Supplement: S8 Fig — Complementary to Results, Fig 5 containing predictions for the remaining models. Simulations with two cells contracting at 50%. Cell centers are separated by 6rc, where rc is the undeformed cell radius. (a-b) densification ratio of triangular elements (color plot) in deformed networks (d-f) tensile stretches and (g-k) compressive stretches of deformed fibers. (m) Orientation distribution of fibers under tension (stretch λ > 1) within the intercellular region across all models. Each violin corresponds to each one of the models studied and shows the distribution of fiber horizontal direction (in degrees), ***p − value < 0.001. Colorbars: (a-c) densification ratio of the deformed networks, (d-k) fiber stretch. (TIFF) [file pcbi.1012238.s008.tiff]

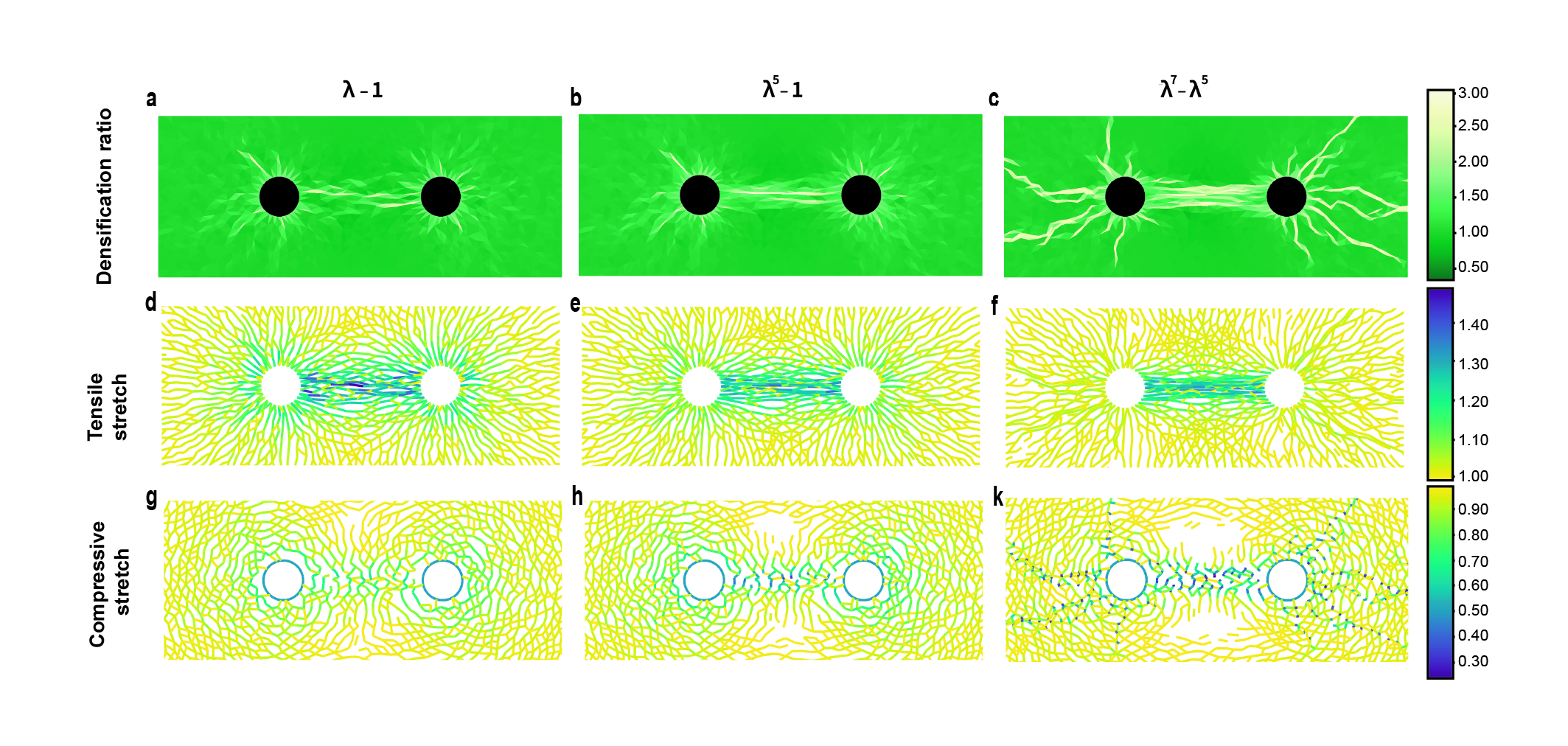

Supplement: S9 Fig — Simulations with two cells contracting at 50%. As in S8 Fig except that cell centers are separated by 4rc, where rc is the undeformed cell radius. (a-b) densification ratio of triangular elements (color plot) in deformed networks (d-f) tensile stretches and (g-k) compressive stretches of deformed fibers. We observe densification around each cell boundary, which extends towards the neighboring cell. Tethers are rather weak for Family-1 cases (a-b) and significantly stronger with Family-2 (c). Within tethers, densification ratio is three times larger than the rest of the matrix. In the intercellular region, fibers under tension are directed towards the neighboring cell so that they form continuous paths connecting the two cells. In these paths, fibers under tension are almost perfectly aligned with the horizontal line connecting the two cells. In Family-2 case (f) excessive tensile stretches are concentrated only within the tether-region. Severely compressed fibers (g-k) locate in the intercellular domain, being roughly perpendicular to fibers under tension. Colorbars: (a-c) densification ratio of the deformed networks, (d-k) fiber stretch. (TIF) [file pcbi.1012238.s009.tif]

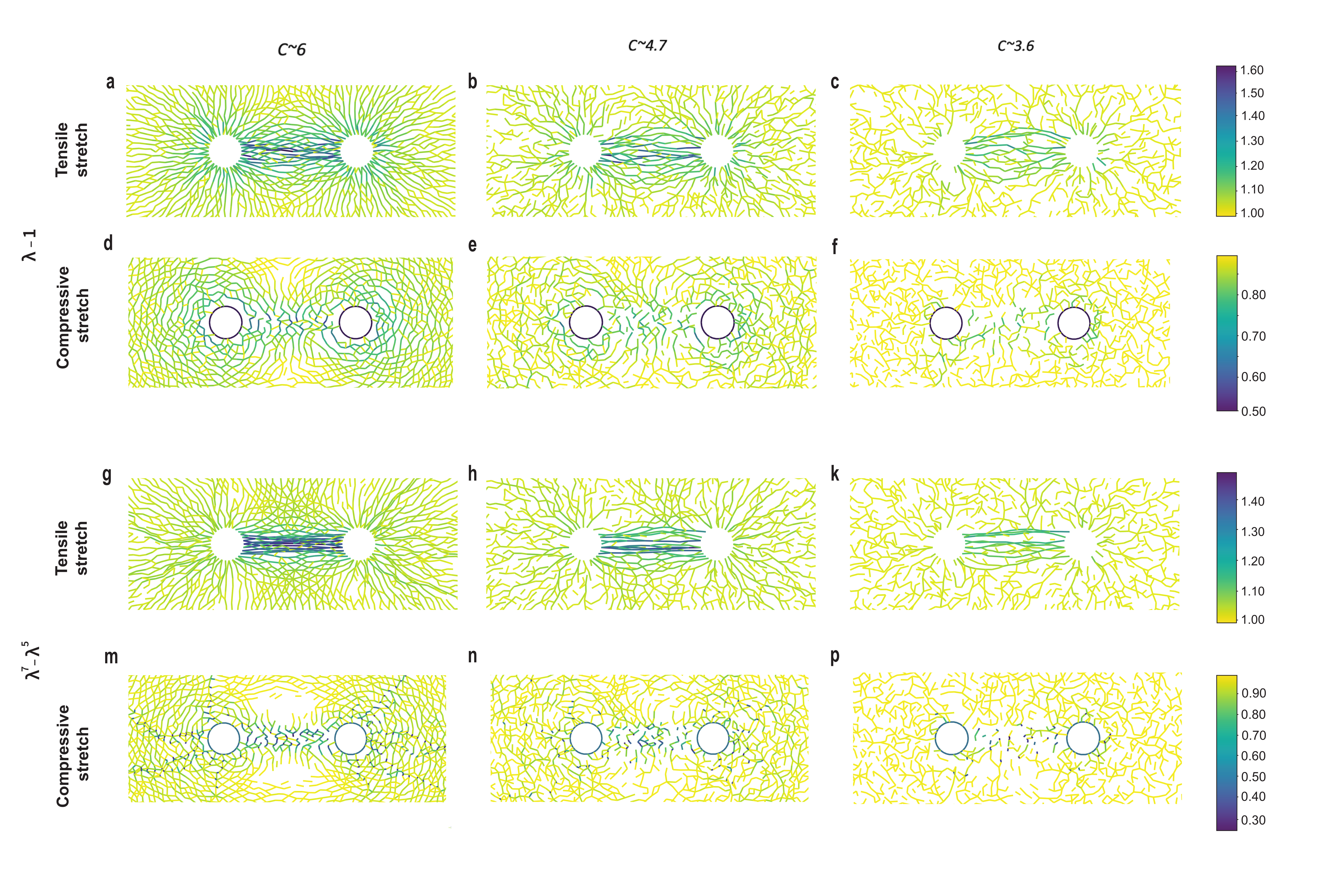

Supplement: S10 Fig — Simulations with two cells contracting at 50% with Family-1 model λ − 1 (a-f) and Family-2 model λ7 − λ5 (g-p). Cell centers are separated by 4rc, where rc is the undeformed cell radius. We examined how decreasing the network connectivity (C) impacts the alignment of fibers between adjacent cells. We report on simulations with C ∼ 6 for a fully connected network, C ∼ 4.7 or 3.6 for networks with lower connectivity. A direct observation of these simulations is the persistence of fiber alignment with the Family-2 model, even at the lowest connected network (C ∼ 3.6). Moreover, expanding upon the primary observation in fully connected networks, where fiber alignment coincides with fiber compression, we observe that this phenomenon persists in less connected networks. This holds for both Families. Despite alterations in geometry, our findings indicate that compression instability facilitates fiber alignment. Colorbar: stretch of deformed fibers. (TIF) [file pcbi.1012238.s010.tif]

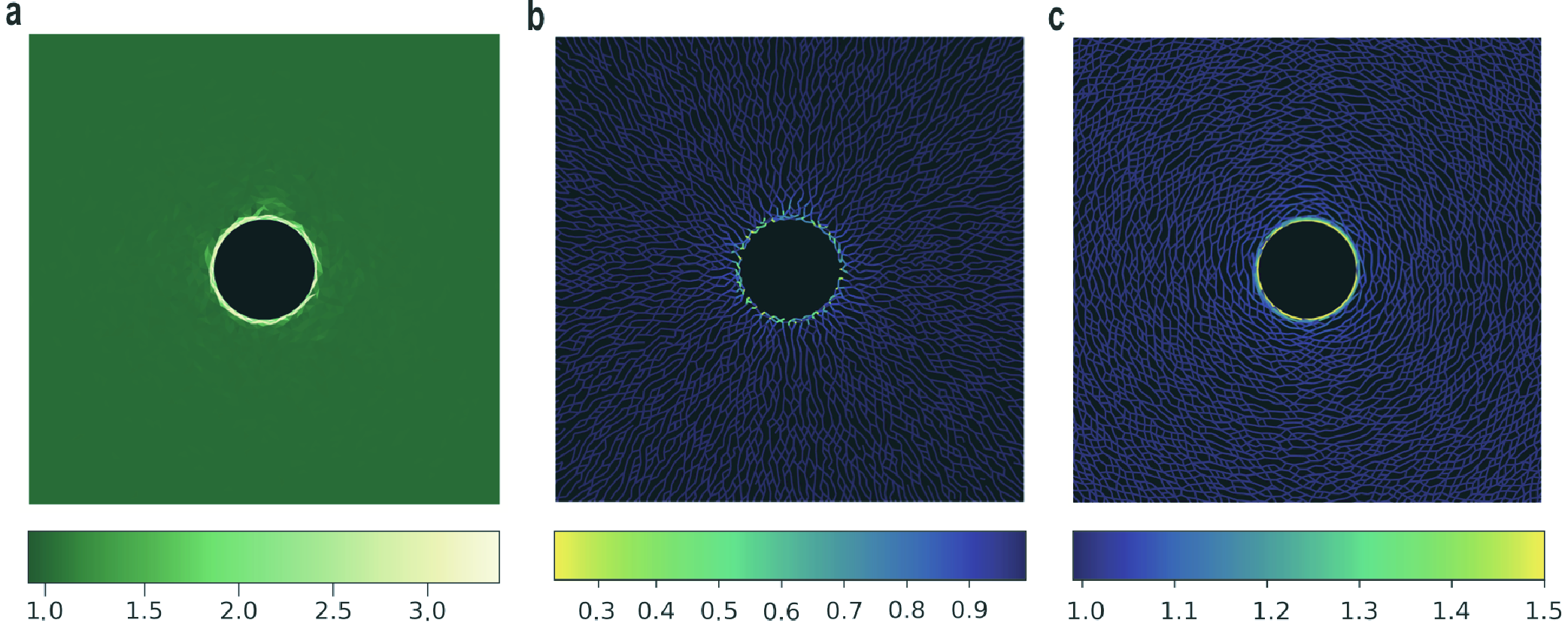

Supplement: S11 Fig — Simulation with S(λ) = λ5 − λ3 of a single cell radially expanded by 50%.(a) Densification ratio of triangular elements (color plot) in deformed networks (b) compressive stretches and (c) tensile stretches in deformed fibers. Note that the compressed fibers align with the radial direction while fibers under tension orient in the angular direction. (TIF) [file pcbi.1012238.s011.tif]
