# Supplementary material for: Compressive instabilities enable cell-induced extreme densification patterns in the fibrous extracellular matrix: Discrete model predictions
Source: PLoS Comput Biol. 2024 Jul 1;20(7):e1012238. doi: 10.1371/journal.pcbi.1012238 (PMC11244807; doi:10.1371/journal.pcbi.1012238)
Supplement: Attachment — Submitted filename: plos_peer_review.pdf [file pcbi.1012238.s013.pdf]

The authors have done a good job in revising their paper. I especially appreciate the more in-depth explanation on the stability of buckling.
However there is still one major point I would like to have clarified: the interpretation of previous experimental work and how it relates to the model.

1) To make sure we are on the same page, I have included a sketch:

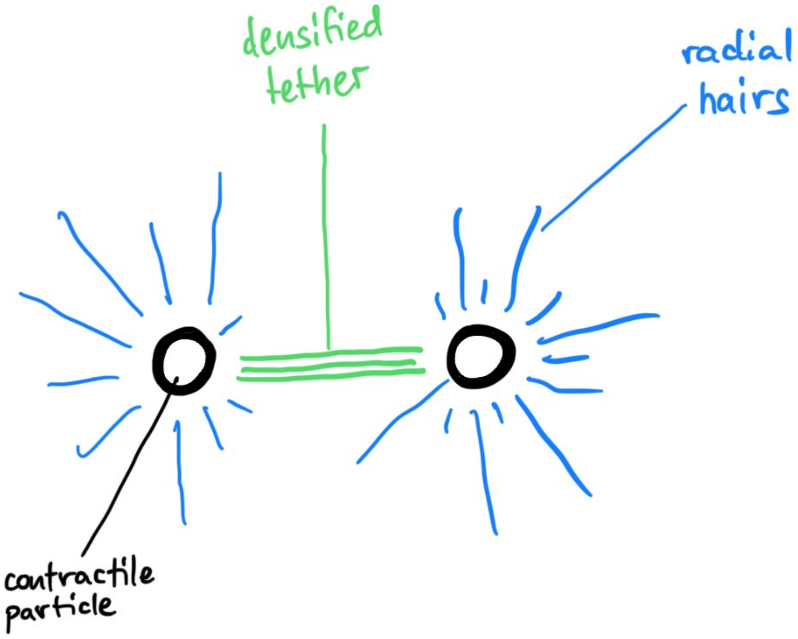

In this sketch, two contractile particles (cells or artificial particles; black) are connected via a densified tether (green). In addition, following the terminology suggested by Grekas et al 2021, the thin densified bands around the contractile particle are “radial hairs” (blue).

I fully agree with the authors that a densified tether is seen in experiments, both with single cells, multicellular aggregates, and artificial contractile partiles as used by Grekas et al 2021.

However, I disagree on the extent of radial hairs in experimental data, particularly at the length scale of cells.

> **In order to rule out such causes as migration or filopodial extension, we used artificial contracting particles in Grekas et al 2021, and found heterogeneous densification patterns as well, see Fig 4a, 4d, 5b, 6b in that paper, where hair-like microstructures emerge around contracting particles.**

Closely looking at the figures that the authors mentioned (**Fig 4a, 4d, 5b, 6b in Grekas et al 2021**) I see a homogeneous “halo” of radial densification around the circumference of the artificial contracting particle that does not spread very far into the ECM, at most up to 1 diameter, while in simulations the radial hairs are inhomogenously distributed along the circumference of the particle, and spread quite far, clearly at least two diameters, into the ECM (e.g. comparing Figure 5b and c in Grekas et al 2021).
Compared to these experimental results, the data of family 1 models in the submitted manuscript, particularly Figure 3a-b visually resemble the experimental data in Grekas et al 2021 the most. Very long radial hairs, as displayed by family 2 models (e.g. Figure 3c), do not seem to occur at the cell scale.

> **Furthermore, this is also clearly explained in Grekas et al 2021 as a scale effect. The scale of multicellular aggregates is larger than the scale of single cells and the width of the thin hairlike bands emanating from them, hence multiple hairlike bands are**

observed emanating from larger clusters or artificial robotic cells (particles) Figs 1c,4a,4d,5b,6b in Grekas et al.

My argument is that the pattern of radial ECM densification in experiments at the scale of single cells resembles the pattern of family 1 models. The very long and inhomogeneous radial hairs of family 2 models do not resemble the data at the cell scale.

In contrast in Notbohm et al. 2015 in experiments with isolated fibroblasts, a single densified tract joining adjacent cells appears immediately upon cell contraction, and hours afterwards filopodia extend slowly along the densified tract; Fig. 5 Notbohm et al. 2015.

The cited experimental work in Notbohm et al 2015 shows an earliest timepoint at 5 hours, not immediately upon cell contraction, and the GFP marker shows filopodia already at this timepoint that clearly overlap with the location of the radial hairs (and also along the tether). From these data it cannot be excluded that the radial hairs in this experiment formed due to filopodial activity.
This should be corrected in the discussion in line 669-671.

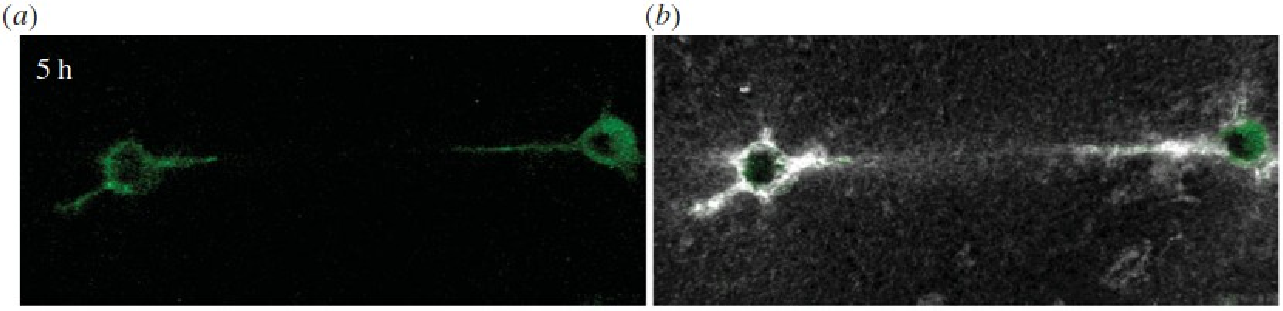

Figure 5 from Notbohm et al. 2015

Minor comments:

1) Line 57: What does “the latter” refer to?

2) The paragraph from lines 59-80 would benefit from clarification that the mentioned “fibers” are not biopolymers, since the references are from the engineering and mechanics literature. For example, the concluding statement (lines 76-80, “These studies strongly suggest that the mechanics of single collagen fibers...”) can be misread to imply that measurements were done on collagen fibers.

3) Line 101: What is “buckle collapse”?

4) Line 177 is the first mention that stiffness is equivalent to the slope of the force-stretch relation. I suggest to mention this earlier, e.g. after Equation 2 is introduced, to make it easier to understand for readers from a biology background.

5) Line 421: “not” should be “note”

6) Line 500: “Most ECM fibrous networks have connectivity that is between 3 and 4” requires a citation.

7) Line 505: “S9 Fig.” should be “Fig. S9”

8) Line 611: Remove “refs”

9) Line 642: “but resulting densification” → “ but the resulting densification”

10) Lines 642-643: “much sharper delineated” → “much more sharply delineated”

11) Line 675: “philopodia” → “filopodia”

12) Line 678: “displacments” – “displacements”

13) Line 679: What does “these works” refer to?

14) Line 680: “philopodia” → “filopodia”
