# Supplementary material for: Compressive instabilities enable cell-induced extreme densification patterns in the fibrous extracellular matrix: Discrete model predictions
Source: PLoS Comput Biol. 2024 Jul 1;20(7):e1012238. doi: 10.1371/journal.pcbi.1012238 (PMC11244807; doi:10.1371/journal.pcbi.1012238)
Supplement: Attachment — Submitted filename: Author Response to Reviewer Comments.pdf [file pcbi.1012238.s012.pdf]

Author Response to Reviewer Comments

Note: Responses to Reviewer Comments are in ***BOLD ITALICS***

Reviewer #1: In this work, Kalaitzidou et al. developed a nonlinear fiber network model of fibrous collagen ECM. Fibers are modeled as the edges of a triangular mesh and two families of models are simulated for evaluating the role of instabilities in ECM remodeling. They provide simulations that demonstrate spontaneous ECM remodeling due to cell contraction.

Overall, the model and results are interesting. However, there are various concerns that prevent me from recommending the paper for publication in its current form. I have detailed these concerns below.

We appreciate the reviewer's insistence on clarity and objectivity, as well as reminding us of the likely difference of culture between authors and readership of the Journal.

1. In the abstract, the authors claim, "This has been avoided so far...". What has been avoided? Understanding the biomechanics of ECM, or fully accounting for nonlinear instability?

Accounting for nonlinear instability has been avoided. In order to clarify it, we have changed the sentence to "by fully accounting for nonlinear instability, which has been avoided so far because it introduces difficult challenges in computation."

2. In the abstract, authors say that they use "cartoon-like models". Do they mean simplified use-cases?

"Cartoon-like models" refer to simple mechanics theoretical models. We have changed it accordingly.

3. In the abstract (and similarly in the author summary), the authors state "remodelling creates complex patterns, comprising narrow localised bands of severe densification and fiber alignment, extending unusually far into the ECM...". It is unclear what the authors describe as unusual, whether the extent of densification is unusual as observed in experimental observations or in the predictions of their model.

We described as "unusual" the extent of densification as observed in experiments. We have changed it to "substantially" , in order to clarify it.

4. In the abstract, the authors describe their model as "fully nonlinear". It is unclear what "fully" vs. "partially" nonlinear means and what features of the model make this claim true or defensible.

"Fully nonlinear" means that we allow both for nonlinearity in individual fiber force-deformation relations and we do not employ small deformation assumptions used to approximate nonlinearities due to possibly large rotations by linear relations. We thus allow full constitutive and geometric nonlinearity and do not use linearisation approximations that can be disastrous for modelling of soft matter, which can undergo very large deformations which require a nonlinear treatment. There are models that linearise the deformations and rotations only; these are partially nonlinear. They can miss many interesting phenomena.

5. The content of the abstract is not well organized. For example, the authors refer to justifications of their modeling assumptions without describing their assumptions. They also jump around a lot between what is observed, what others have done, and what they did in this work.

Please see the revised abstract, we feel it has been improved. Our assumptions include single fiber unstable buckling. See also the new paragraph on buckling p.88 that clarifies things further.

6. In the abstract, the authors claim that no other model "provides a simple mechanism of compressive instabilities that fully explains the phenomenon". It's unclear what they mean by "fully explain". Presumably they don't mean that their model explains everything involved in densification, fiber alignment and emergent patterns.

We agree that this is too much, although our model does predict all these 3 phenomena. We have changed the sentenceto "Previous work does not capture the [...]. Our model shows that these phenomena can be explained by simple mechanisms of compression instabilities."

7. In the abstract, the authors claim that their model is "simple and easy to comprehend". This claim as written entirely depends on the knowledge and expertise of the reader and so I don't think that it is an appropriate claim to make without some justification or clarification.

We agree. Thus, we changed it to "Our model is simple, but manages to describe a very complex multi-stable energy landscape, [...]".

8. In the abstract (and similarly throughout), the authors describe "striking patterns of ECM pathways" and claim that their model predicts them but provide no quantitative metrics to judge how well such predicted patterns match those observed in any experiment or justification as to why qualitative assessment is sufficient. This diminishes the reproducibility and credibility of the work, and the authors should address this by either providing such metrics or, in the absence of any established metrics to do so, should state as such.

Our model predicts (recapitulates) the special morphology of densification patterns qualitatively. We feel that is no issue with reproducibility of the model's predictions. It also predicts for Family 2 tether formation at the physically observed level of cell contraction (about 50%) Other models require much larger values like 80% that is not observed in experiments. Our model also predicts the roughly 3-fold increase in density within densified zones observed in experiments (Grekas et al. 2021 among others). It is very difficult to make more quantitative predictions as more quantitative data from most experiments are not available.

9. Similarly, in line 413 the authors claim that a solid tether forms and then further develop with this concept in the following paragraph (e.g., Figure 6). It's unclear what constitutes a "solid tether" vs. "not a solid tether" besides a qualitative assessment, which also diminishes the reproducibility and credibility of the work. Later (line 497) the authors somewhat define tethers, though qualitatively, as "zones of high densification".

Tethers are defined in the very beginning of the Results: Intercellular tether formation in two-cell simulations. In particular, line 432-433 revised manuscript, tethers are defined as roughly parallel bands of high densification and fiber alignment, that connect two cells. The term "solid tether" simply means a continuous (unbroken) tether joining cells continuously. The term has been changed to "a tether that extends continuously from one cell to the other".

10. In the author summary (lines 5-6), the authors claim that "Observed response of the ECM to forces from contracting cells is unexpected". By response, do they mean mechanical or chemical? One would obviously expect a mechanical response by fibrous ECM to externally applied forces, but also previous models of other ECM components have demonstrated responses like fibronectin fibrillogenesis (https://doi.org/10.1038/s41598-017-18328-4) to externally applied forces.

Mechanical response is meant here. In Grekas et al [18] we showed experimentally by using active contracting spherical particles instead of cells, that densification patterns emerge immediately upon contraction of these particles, without any chemical involvement and with no time for chemical diffusion to occur at such large distances (often more than 10 particles diameters). We mention this in various places Chemical remodelling of the ECM is important but beyond the scope of this paper.

11. In the Author Summary (lines 10-12), the authors state that they "hypothesise that this behaviour is caused by the inability of fibers to resist compression, much like rubber bands, and the fact that in a network, there is room for fibers to rotate, buckle and collapse". Which behavior(s) is the authors hypothesizing is due to these fiber characteristics?

This was clarified; see red text in summary.

12. Figure 7 subpanels a and b need labels on their vertical axis.

Corrected.

13. In line 559, the authors refer to a compression instability being "activated" but it is not unclear what exactly is an "activated compression instability".

This refers to the system undergoing a transition from stable equilibrium to an unstable state, like a fiber starting to collapse under compression or like a group of fibers starting to collapse through stap-through buckling (element collapse). These are explained and described in Methods; see Nonconvexity and Instability.

14. In line 570, the authors claim that their "results show that there is a single unifying mechanism behind densification, fiber alignment and matrix anisotropy, ...". Certainly, they have shown that this is true for their model. But the wording reads as if the authors are claiming that their results show that this is how we should understand the mechanisms responsible for densification, fiber alignment and matrix anisotropy. Either this language should be refined to narrow the scope of the claim, or some justification should be made as to why other mechanical and chemical features of the ECM neglected by their model are unimportant to our understanding of these phenomena.

"Single" here refers to one mechanism that explains three phenomena simultaneously. We never claim that there are no other candidates for this capability. Alternatives may be there and this is up to other researchers to investigate. We discuss some alternative models in lines 666-697 (revised) We never exclude other possibilities, but merely show that a largely ignored mechanism does the job

remarkably well. We never say that it it the only one that does. One can never show that conclusively. We agree that's the wording can be misunderstood and have softened it. (line 649)

15. The authors describe in line 550 that "unreasonably high cell contraction levels" were simulated and reported in the work and refer to "experimentally observed cell contraction levels" in lines 552-553. It's unclear how to interpret simulation results, whether as biologically or only theoretically meaningful.

We do interpret simulation results and observe that Family 2 models produce densification patterns at contraction levels consistent with experiments (typically 40-50%) whereas Family 1 models require much higher contraction levels, often as high as 80% radius reduction which are not encountered experimentally. We interpret this as evidence that family 2 is a better model. We state: Our work shows that instability can occur even in models with stable stretch response (Family 1), but it does so at cell contraction levels that are substantially higher than experimentally observed ones. This casts doubt on the biological relevance of Family 1, and prompts us to introduce models (Family 2) whose stretch response becomes unstable in compression.

16. Given that simulations produced fibers that nearly collapsed (e.g., line 304), the sensitivity of results to the parameters "Q" and "b" in Equation 8 seems important. However, the authors do not show whether either parameter has any effect on results.

We added a figure to discuss this S8 Supplementary Figure and a statement following Eq. (8). The term involving Q and b prevents total collapse of fibers into each other and fibers going through each other (interpenetration of matter) as explained in Methods: **Energy Penalty for Interpenetration of Matter .** ***Once this is achieved the detailed values of Q and b do not greatly affect densification morphology. Decreasing b slightly increases density within tethers.***

Reviewer #2: Understanding how cells mechanically interact with the extracellular matrix is a hot topic that has relevance for many fields in biology. In this manuscript, the authors develop a discrete fiber network model to describe extracellular matrix mechanics, and investigate how a circular inclusion mimicking a contracting cell deforms the matrix. They construct a network made of triangular elements, and compare two different families of constitutive models for the force-extension behavior of individual fibers (sides of the triangles). Both families of models account for stiffening in tension and softening in compression, while family 2 additionally accounts for non-linear behavior ("stretch-instability") in compression.
The manuscript is very well written, with clear and polished language. The results are interesting and explained well. The authors give a good broad introduction and discuss large parts of the relevant literature. Some of the claims are stated too broadly, and some points need to be clarified further, which the authors can address with a minor revision.

We thank the Reviewer for positive remarks and thorough comments.

%%%% Major comments

1) The authors claim that their previous continuum model (reference 18) and their new discrete model in this manuscript predicts thin hair-like densification which they describe as "localized densification patterns that bear a strong resemblance with experiments [13, 16, 18]." (lines 56-57). However, the cited literature shows these patterns only in multicellular aggregates, where it is unclear if they emerge purely due to mechanics or due to cell migration or extension of filopodia into the tethers.

In order to rule out such causes as migration or filopodial extension, we used artificial contracting particles in Grekas et al 2021, and found heterogeneous densification patterns as well, see Fig 4a, 4d, 5b, 6b in that paper, where hair-like microstructures emerge around contracting particles. Furthermore, this is also clearly explained in Grekas et al 2021 as a scale effect. The scale of multicellular aggregates is larger than the scale of single cells and the width of the thin hairlike bands emanating from them, hence multiple hairlike bands are observed emanating from larger clusters or artificial robotic cells (particles) Figs 1c,4a,4d,5b, 6b in Grekas et al. In contrast in Notbohm et al. 2015in experiments with isolated fibroblasts, a single densified tract joining adjacent cells appears immediately upon cell contraction, and hours afterwards filopodia extend slowly along the densified tract; Fig. 5 Notbohm et al. 2015.

Experimental work with single rounded cells does not seem to have these extremely localized heterogeneities, unless filopodial protrusions are included (see e.g. Mann et al. 2019 [reference 38 in the manuscript], Malandrino et al. 2019, Gomez et al. 2019 [reference 56 in the manuscript]).

This issue was addressed in Notbohm et al. 2015. There it is shown from experiments with isolated fibroblasts, that densified tracts appear immediately upon cell contraction, and AFTERWARDS filopodia extend along the densified tract; Fig. 5 Notbohm et al. 2015.

Some of the authors' previous experimental work using synthetic contractile particles also lacks the thin highly heterogeneous densification bands (Grekas et al. 2021 [reference 18]).

We do not understand this comment. In Grekas et al., there are multiple examples of the "thin highly heterogeneous densification bands" both in experiments with synthetic particles and in simulations of the continuum model. See in particular Figs 1c,4a,4d,5b, 6b. All these bands appeared upon contraction of the robotic particles, which are incapable of filopodial protrusion. Also in SHih et al. Supplemental Videos It is clear that first adensification band forms between multicellular acini while they contract, and THEN individual cells start migrating along them

This would suggest that their family 1 model might be a better suited for single cell force-transmission, while their family 2 model might better represent matrix deformation by multicellular aggregates.

The authors should revise the statements in the introduction and extensively discuss this point.

We feel that this cannot be the case, because the mechanical behaviour of single fibers (thus the model choice of Family 1 or 2) is totally independent of whether it is a single cell or an aggregated that deforms the matrix. The scale of inhomogeneities here is set by the fiber size versus the cell or aggregate diameter. This is explained carefully in Grekas et al 2021,

Malandrino, Andrea, et al. "Dynamic filopodial forces induce accumulation, damage, and plastic remodeling of 3D extracellular matrices." PLoS computational biology 15.4 (2019): e1006684.

We feel that the above paper is complementary to our work and they do not seem to contradict each other. Filopodial extension is beyond our scope. The experiments in Grekas et al. using robotic cells (active microspheres that contract on demand ,embedded in fibrous collagen, and cannot produce philopodia) show densification bands and tethers like the ones observed here   Induced by particle contraction.   Also the model used there and in Tsingos is linear and whether or not buckling is observed depends on the choice of stiffness constants, so the conclusion in Tsingos et al. that no buckling is observed may be due to the overly stable model.
These papers are discussed briefly in lines 666-675

2) One central claim is that the authors' model is unique in capturing long-ranged and spatially heterogeneous densified bands of extracellular matrix around a contractile inclusion. It is true that most discrete fiber network models have very homogeneous deformations around the inclusion, but there are examples of heterogeneous deformation, e.g. for some network geometries (Humphries et al. 2017), and for networks with low connectivity (Slater et al. 2021, Ruiz-Frano and van der Gucht 2021, Tsingos et al. 2023). This previous work should be acknowledged and discussed. It would be interesting to highlight qualitative differences between the densification heterogeneity observed by the authors and by previous work.

We do not claim uniqueness. It is impossible to predict the efficacy of future models, which hopefully will do better than ours. We claim that among existing models that attempt to predict densification patterns like the ones observed in experiments, ours is very successful, typically more so than some previous work, with notable exceptions discussed in lines 690-697. Also the two distinct instability mechanisms we identify here (as opposed to one in our previous work, provide a unified explanation of the appearance of densification, pattern formation and fiber alignment. We do never claim exclusively.

Clearly network geometry will affect ECM deformation. Low connectivity in effect introduces inhomogeneities in network fiber density, which inevitably result in deformation inhomogeneities. In a continuum analogue of a low connectivity network, one would introduce random holes which would cause inhomogeneous deformation even under homogeneous forces. There is no surprise here from a mechanics point of view. On the other hand, the new instability mechanisms identified in our work are active even in perfectly homogeneous regular networks, and this is new and surprising. See lines 675-689.

We never claim that instability is the only mechanism, but our work clearly shows that it is highly relevant because of its predictions, in a way that was not recognised before.

Humphries, D. L., J. A. Grogan, and E. A. Gaffney. "Mechanical cell–cell communication in fibrous networks: the importance of network geometry." Bulletin of mathematical biology 79 (2017): 498-524.

This is discussed, see above

Ruiz-Franco, José, and Jasper van Der Gucht. "Force transmission in disordered fibre networks." Frontiers in Cell and Developmental Biology 10 (2022): 931776.

This paper studies force chains which are interesting but not necessarily directly related to densified bands. We also had to exclude some of the dozen papers suggested by the three reviewers

Slater, Brandon, et al. "Transient mechanical interactions between cells and viscoelastic extracellular matrix." Soft Matter 17.45 (2021): 10274-10285.

This paper is somewhat beyond the scope of our work as it focuses on time dependent viscoelastic regimes.

Tsingos, Erika, et al. "Hybrid cellular Potts and bead-spring modeling of cells in fibrous extracellular matrix." Biophysical Journal (2023).

This is discussed, see above.

3) Related to point 2. Fiber densification: The authors define fiber densification by taking the ratio of the area of each triangular element to its undeformed reference area. In experiments, typically the density of fibers per unit area is quantified. The authors should verify (numerically) that these two metrics are indeed directly comparable.

To be precise we define densification ratio, not fibre densification ratio in a way consistent with both continuum mechanics and discrete network mechanics, as it provides information about mass density changes in view of mass balance. It also provides very useful information about densified regions because it predicts the collapse of fibers and fiber elements in view of elementary triangle geometry. The number of fibers per unit volume is somewhat vague and not universally used in experiments. In experimental data density is measured by estimation of the displacement gradient. There, beads are placed within the matrix and the displacement field of the beads is estimated, generating an estimation of a the deformation gradient. The definition used there is consistent with ours and with mass balance.

4) Related to point 2. What is the average connectivity of the networks the authors studied? For reference, average connectivity in collagen matrices is between 3 and 4 (Jansen et al. 2018, Burla et al. 2020).

It is around 5.5-6. Here following your comment we have now included new computations of tether formation between two cells in low connectivity networks; see lines 499-505 and supplemental fig. S9.

However, we note that low connectivity in effect introduces inhomogeneities in network density, which inevitably result in deformation inhomogeneities. In a continuum analogue of a low connectivity network, one might introduce random holes which would cause inhomogeneous deformation even under homogeneous forces. There is no surprise here from a mechanics point of view. On the other hand, the new instability mechanisms identified in our work are active even in perfectly homogeneous regular networks, and this is new and surprising. See also lines 675-689 for a related discussion.

In earlier work Notbohm et al. 2015 we had found that low connectivity does not cause extensive densification by itself IN THE ABSENCE of buckling

Burla, Federica, et al. "Connectivity and plasticity determine collagen network fracture." Proceedings of the National Academy of Sciences 117.15 (2020): 8326-8334.

The work by Burla et al. addresses a different phenomenon, namely fracture, and while very interesting, is not directly relevant here.

Jansen, Karin A., et al. "The role of network architecture in collagen mechanics." Biophysical journal 114.11 (2018): 2665-2678.

That work does not study the effect of network geometry on the inhomogeneous deformations studied in our paper. It considers specialised deformations (shear) because of their occurrence in rheometer experiments. The scope is different from our work.

5) Line 547: The authors claim that previous discrete network models of the matrix do not address instability. If the authors refer to buckling, then the statement is wrong, since others have investigated the effect of buckling, e.g. Notbohm et al. 2015 (cited as reference 17). The authors should clarify what they mean by instability and revise the statement accordingly.

Instability refers to the existence of unstable equilibrium of the system (the smallest perturbation will take the system away to another stable equilibrium). Instability is closely related with loss of convexity of the energy function of the system, as discussed extensively in Methods: Nonconvexity and Instability.
Buckling is not synonymous with instability. Please see blue paragraph lines 88-105. There is stable and unstable buckling. In the former the system can smoothly and gradually buckle as forces are increased. A single family-1 fiber is an example. In unstable buckling the system has to dramatically jump over unstable states to a far-away stable equilibrium, for example a fiber of Family 2 type will collapse suddenly. We identify TWO instability modes for fiber networks here. One of them is related to unstable fiber buckling (Family 2). This was not done before for biological materials , except in our own work here for discrete network models and in Ref.18 for continuum models of collagen fiber networks). The second occurs even when the fibers are stable but whole elements (larger fiber groups, triangles in our model) suddenly collapse. We have a whole section in Methods: Nonconvexity and Instability. where these are described and explained in detail with a clear definition of instability.

Notbohm et al 2015 (using the idea from Notbohm's PhD thesis 2013) is indeed the first paper to propose buckling as a mechanism of densification band formation in a biomaterial. However the bands occurring in this model are much more diffuse than observed because the model used is a Family 1 type model (stable buckling) that forms weaker tethers as shown here and also because small deformations were used in the simulations. Similar comments apply to other previous work, see lines 636-644

6) It is not clear what the authors mean by "rotations". Are these rotations of individual fibers, of triangles, or of the order of vertices in a triangle (clockwise vs counterclockwise)? Related to this, the authors claim that previous models are restricted to small rotations, but it is unclear what is meant. Please explain.

It refers to rotations of fibers. Typically in the engineering theory of trusses, which is related to fiber network models, it is assumed that displacements of the nodes (fiber endpoints) are small compared to fiber length. This in turn implies that fiber rotation angles of fibers are small which allows one to eliminate nonlinearity in the equilibrium equations by approximation (for example for a rotation angle θ one approximates $\sin\theta$ by transverse relative displacement of fiber ends divided by undeformed fiber length.) This type of approximation (linearisation) yields a linear system of equations that can be solved by linear algebra methods, however, it also throws out instabilities such as buckling or snap-through considered here (both fiber and element collapse instabilities). Some investigators use nonlinear stress-strain relations for fibers but small rotations and some readymade codes do that automatically . The results cannot account of many instabilities like snapthrough considered here

***(element collapse instability)*.**

%%%% Minor comments

It would be interesting for the authors to quantify the force transmission distance, as this would allow them to compare their data to previous work. See section 4.3 of the following paper for an explanation.

The paper mentioned by the reviewer mentions displacement decay. So we added extensive computation results which quantify the displacement spatial decay away from a single contracting cell and compare results with other models; see lines 396-428 and Fig.5 (new). We observe that Provenzano et al. shows that alignment of densified fibers guides migrating cancer cells even after fiber forces have been removed by thermal treatment. Alignment extends at large distances so long range displacements are quite possibly relevant for migration and metastasis.

Wang, Haiqin, and Xinpeng Xu. "Continuum elastic models for force transmission in biopolymer gels." Soft Matter 16.48 (2020): 10781-10808.

Line 201: Typo, "neighoring" should be "neighboring"

Line 206: Unexpected newline

Line 207: Sentence should end with a full stop.

Line 288: "continual" should be "continuous"

Line 453: Typo, "Our simulations results" should be "Our simulation results"

Line 524: The word "offer" doesn't fit into the sentence.

All the above have been corrected.

Reviewer #3: The study introduces a model that explains how fibrous collagen matrices change when cells exert mechanical forces. This leads to patterns of densification and alignment, important for cell movement, invasion, and tumor behavior. This model considers fiber instability and rotations, causing compression instabilities like buckling. Due to concerns explained below, it is difficult for me to recommend this article for publication.

We thank the reviewer for criticisms that prompted us to (hopefully) improve the paper and to further clarify ideas and explanations.
< Major >

- The family-1 model is based on decreasing stiffness of fibers with increasing compression. Ref. 33 that the authors cited already demonstrated the importance of buckling in a more straightforward way by putting two segments between adjacent nodes of the lattice structure. Using the model, it clearly showed the role of buckling in long-range force transmission and remodeling of ECM. Figs. 3A, B in the paper show a difference between cases with or without buckling. Fig. 4G shows the bundle formation between contracting units in the presence of fiber buckling.

Ref.17, Notbohm et al. (with results from Notbohm's thesis 2013) was perhaps the pioneering work proposing and showing that buckling of individual fibers plays an important role in densified-zone formation in fibrous biomaterials, such as collagen ECM. This precedes Ref. 33. The first study to identify buckling as an instability in elastic networks was Ref. 43 back in 1993. It was great to see that Ref 33, a model with actual buckling represented by segmented fibers, confirmed some of the conclusions of Ref.17. Ref. 17 used a model for buckling that is more minimal, but captures many aspects of the buckling response of a stiff rod with two segments connected by a rotational spring. In particular, it is possible to compute the postbuckling relation between effective length (distance between endpoints) of a buckled rod as in ref. 33, and compressive force at the rod ends. This was done in Ref. 31, Fig 1a (without being mentioned explicitly) and it is qualitatively similar to Fig 1b,in ref. 17, the bilinear model in Ref. 17. So in essence lower stiffness in compression. The lower stiffness in compression is meant to represent the postbuckling behaviour of force versus effective length, or the distance between fiber ends. The idea of effective length is made clear in the present paper under review, at the beginning of section 2.1 and in Fig. 1b. It allows us to simplify the geometry of buckling so we can focus on effective mechanical properties and energetics of buckled fibers.

After Refs 17,31, we discovered (Grekas et al. Ref. 18) that there is more to the story. In particular, the continuum model we deduced by averaging fiber properties, surprisingly showed nonconvexity of the energy landscape. This entails loss of stability as in phase transformations. Instability had not been considered before in the context of bio-ECM, including in our own work 17,31. Instability really changes the game for continuum models because it results in spontaneous pattern formation with localised densification bands, Ref.18, as seen in experiments. Such pattern formation is forbidden in stable continuum models of linear or nonlinear elasticity. This motivated us to go back to the discrete model in this paper and to study stability, finding that it fails even for networks of linear fibers that do not buckle, provided one allows large enough deformations with geometrically exact rotations. This "new" instability involves snap-through collapse of larger fiber groups as opposed to single fibers. Also it occurs in Family-1 networks with stable single fiber response, as well as in Family-2 networks. This means that instability comes in more than one flavour and is probably ubiquitous in fiber networks. In all cases, these instabilities require compressive stretches and result in localised densification zones.These results are new and clearly separate this work from previous ones. Please see lines 88-105 for explanations.

Thus we do not only consider fiber instability, but also the effect of rotational invariance of fiber energies, which causes nonconvexity of the energy landscape, Fig. 2a. This results in a different, additional instability mechanism, familiar in the nonlinear truss literature, but ignored so far in fiber network mechanics. We show that all these instabilities have a significant effect in fiber network mechanics. While fiber buckling has been considered before Refs 17,18, 33, and others, the role of rotations and the role of multi-fiber element collapse instability have not been considered before in the biomechanics literature. So densified zone formation does not need fiber buckling but can be caused by element snap-through buckling collapse of fiber triangles, although our results show that this occurs at very large cell contractions, typically above physiologically observed levels.

- The family-2 model is based on an assumption that stiffness becomes negative at extreme compressions.
This was already considered in a similar way in DOI: 10.1038/s41467-017-01011-7. In their model, if fibers located within a critical distance, the fibers are merged, which is either due to fiber entanglement or chemical bond formation. They showed significant densification and bundle formation between two cells. This looks very similar to the authors' assumption. Although the authors might claim their way is a much more rigorous way, their potential "S" seems to be determined in an arbitrary manner too.

We were not aware of this paper which is highly relevant here, thank you veymuch for bringing it to our attention. The fibers in that paper are linear with positive stiffness. The tether formation relies on additional assumed effects like sliding of cross links ("plasticity")and better known effects like merger of fibers. This is one of the few papers producing tethers as strong as ours. In Grekas et al. 2021 we also use a model with merger of fibers by adding an attractive potential between them. It amplifies the collapse instability and produces even stronger tethers that are irreversible. We discuss this paper in lines 691-698.

- I can see the family-2 model can induce bundle formation at lower contraction strength than the family-1 model. However, this doesn't necessary mean that the fiber instability (negative fiber stiffness at extreme compressions) is actually responsible for the bundle formation in ECM structures. This fiber instability was devised from fundamental mechanics papers (Refs. 43-46). The authors didn't provide any evidence showing this fiber instability exists in collagen fibers.

Many new models involve unproven assumptions to some extent. The real test of such models is whether they predicts observations. Our model predicts extreme densification, severely inhomogeneous localized zone formation (in relatively homogeneous discrete media) strong fiber alignment in densified zones, intercellular tether formation, hair-like microstructures emanating from contracting clusters, <u>slow decay of displacements with distance from the cell added in lines 396-428 and new Fig. 5</u> and other phenomena, all of which have been observed.

Regarding the unproven assumption of fiber instability, one can argue in reverse here. There is no evidence to justify linear force-stretch relations assumed in 33 , and two segmented beams with a rotational spring etc. However, one does not reject them for this, as the model is an excellent idealised model and does capture some behavior, despite ample evidence of nonlinear stiffening in tension. There is certainly no evidence that post-buckling behaviour of fibers is STABLE (e.g. as in Family 1 models).

In fact there is ample indirect evidence to the contrary. Most of it is theoretical or from experiments in similar systems (. It is well known that collagen fibers are hierarchical with complex microstructure. (Piechoka et al 27)
There is ample theoretical evidence Precisely because of instability it would be nearly impossible to obtain experimental evidence.
Buckling of fibers has been shown experimentally. Some studies using linear fiber models do not predict buckling at all (Tsingos et al) and display really weak almost nonexistent tethers. There is no justification for linear models except mathematical ease and tradition, yet no one seems to question them so far. The fact that we are using a new hypothesis on fiber instability that cannot be <u>directly</u> tested experimentally, in no way invalidates our model. There is plenty of indirect evidence that motivates our assumption on instability: Tarantino et al, Shima et al, Piechoka et al. Champneys et al. The real test is whether the new model predicts observations. We present ample evidence that it does so much better than many other models including our own, Notbohm2015, Rosakis2015. We do not deny the role of other mechanisms. We merely show that the instability mechanisms are ubiquitous in networks and are sufficient to predict the phenomenon, while they often do so better than the other mechanisms. It is certainly possible that multiple mechanisms cooperate but it is important to study instability as a candidate first, in a system that is unstable because of network discreteness effects.

Of course we used fundamental mechanics papers to build our model. Mechanics is an essential part of biomechanics. We do not see why this is a problem.

There are many other ways (shown in previous literature) helping the bundle formation, such as breakage of cross-linking points. At least, the breakage of cross-linking points between collagen fibers has been shown experimentally. The fiber instability that the authors focused on in this paper could be just one of the many ways for clear bundle formation. Consider this and the lack of evidence, the authors should not over-emphasize the importance of the fiber instability in the ECM structures.

Lack of direct evidence for a hypothesis of a theory does not disqualify it. What does disqualify it is disagreement of its predictions with experiment. We feel our model is very strong in this direction and competes very well with previous models.

We do not deny the role of other mechanisms. We merely show that the instability mechanisms are ubiquitous in networks and are sufficient to predict the phenomenon, while they often do so much better that the other mechanisms and through use of a simple model. It is of course possible that multiple mechanisms cooperate.

Breakage of cross linking points does contribute to the effect but the work of Vader et al shows that tether formation in fully cross-linked networks is fullly reversible and can occur without fracture or plasticity effects. This foes not mean that they do not contribute in other cases, but they are not necessary for densification patterns.

- Even though this paper considers intercellular tether formation between two embedded contractile cells as one of their crucial findings, this phenomenon seems to have been studied in many papers. Although some of these studies were cited, there should be more thorough comparisons and discussion between this study and the previous studies.

We have been working on this since 2014. So in no way do we consider that tether formation is a (crucial or not) finding of this paper. We think that strong tether formation is a prediction of compressive instabilites not considered before (this does not include stable buckling used by most studies before this one). We provide ample references for tether formation and are happy to include some we have missed, noting that many have also missed or ignored our previous work Grekas et al 2021.

i) DOI: 10.1016/j.bpj.2014.08.029

We site this paper. ***We have added comment in the discussion.***

ii) DOI: 10.1115/1.4026179

This paper is commented on, although we find the active agent model difficult to understand, the results are interesting we discuss it in lines

iii) DOI: 10.1016/j.devcel.2017.12.004

We do not understand the relevance of this paper and do not cite it.

iv) DOI: 10.1038/s41467-017-01011-7

Cited and discussed in Dsicussion lines 691-698.

< Minor >

- What is u_0 from Equation (11)?

It is the prescribed radial displacement of the cell boundary that determines cell contraction.

- The figures are not properly labeled. For example, In Fig. 3m, what is the color bar means? In Fig. 7c and 7d, the color bar has no description.

Explained in captions. If unclear, will be happy to improve.

Have the authors made all data and (if applicable) computational code underlying the findings in their manuscript fully available?

All data will be made available upon acceptance of the paper, or upon reviewer request.
