# Supplementary material for: Compressive instabilities enable cell-induced extreme densification patterns in the fibrous extracellular matrix: Discrete model predictions
Source: PLoS Comput Biol. 2024 Jul 1;20(7):e1012238. doi: 10.1371/journal.pcbi.1012238 (PMC11244807; doi:10.1371/journal.pcbi.1012238)
Supplement: Attachment — Submitted filename: Revision Reviewer Comments & Responses.pdf [file pcbi.1012238.s014.pdf]

PLOS 2nd Revision Reviewer Comments & Responses

Reviewer Comments appear in Roman text. Author responses are in ***bold italics.*** Quotations are in underlined Roman.

==

Reviewer #1: The authors have made significant improvements to their manuscript and addressed most of my concerns.

In their response to reviewers, the authors mention a "likely difference of culture between authors and readership of the Journal". I'm confused about the intent of this statement given that scientists from engineering, medical, and mathematics departments (like the authors) commonly publish in (and so presumably read) PLOS Computational Biology, and about how this statement is relevant to my feedback. I bring this up because of my concerns that were not addressed in revisions, specifically on the organization of content in the abstract and reproducibility of their results. I assume that these concerns were not addressed for other reasons and not because of presumed culture differences but, in case my assumption is incorrect, would like to point out that neither concern is particular to disciplinary background or culture. They are issues of the rigor and potential impact of the work.

We are sorry our comment caused concern. Reviewer 1 (also Reviewer 2) asked us in some comments to explain some of our terminology, which can be unclear to biologists. All these served as a reminder to us that when attempting to publish in a journal from a discipline outside our own, it is important to use language/terminology that is intelligible to readers of the journal. It is often difficult to do this without help from reviewers, who can point out such differences in language, hence our comment. We have made revisions accordingly.

Comment #5: Revisions address detailed concerns in the abstract but do not try to improve the organization of the content. At various times, the abstract jumps back and forth between the innovation, assumptions, and relevance of the work in this project, limitations in other models, motivating biology, etc., in a haphazard way that makes it difficult (at least to me) to perceive the overarching story of the work. I'm disappointed that the authors were not responsive to this feedback, which was intended to improve the likelihood of attracting readers to their paper.

We tried to improve the organization and clarity of the abstract, but apparently fell short regarding the former. We appreciate the Reviewer's high standards of clarity. We modified our abstract and below we break it down, in order to better explain our rationale on organization. We feel that our revised abstract strongly emphasises what Reviewer 1 calls "the overarching story of the work", ***namely that (possibly multiple) instability mechanisms play a crucial role in mechanical remodelling of the fibrous-collagen-network ECM. Please see below.***

ABSTRACT (REVISED)

[bold sentences in brackets are not part of the abstract; they are intended to explain our attempt at organization of ideas. They might be useful to the Reviewer in suggesting further improvements]

[A) One-sentence summary of what phenomenon we study and how]

We present a new model and extensive computations that explain the dramatic remodelling undergone by a fibrous collagen extracellular matrix (ECM), when subjected to contractile mechanical forces from embedded cells or cell clusters.

[B) Brief description of the phenomenon and its unusual aspects]
This remodelling creates complex patterns, comprising multiple narrow localised bands of severe densification and fiber alignment, extending far into the ECM, often joining distant cells or cell clusters (such as tumours).

[C) Importance of the phenomenon]
The importance of these bands is that they serve as pathways for mechanical signalling, cell migration, invasion, metastasis, and are recognised tumour-associated collagen biomarkers.

[D) Shortcomings of previous models]
Most previous models cannot capture this behaviour, as they assume stable mechanical fiber response with stress an increasing function of fiber stretch, and a restriction to small displacements.

[E) Distinguishing features of our model]
Our fully nonlinear network model distinguishes between two types of single-fiber nonlinearity: fibers that undergo stable (supercritical) buckling (as in previous work) versus fibers that suffer unstable (subcritical) buckling collapse. The model allows unrestricted, arbitrarily large displacements (geometric nonlinearity).

[F) Justification of new assumptions]
Our assumptions on single-fiber instability are supported by recent simulations and experiments on buckling of beams with a hierarchical microstructure, such as collagen fibers.

[G) Model implications]
We use simple scenarios to illustrate, for the first time, two distinct compressive-instability mechanisms at work in our model: unstable buckling collapse of single fibers, and snap-through of multiple-fiber groups. The later is possible even when single fibers are stable. Through simulations of large fiber networks, we show how these instabilities lead to spatially extended patterns of densification, fiber alignment and ECM remodelling induced by cell contraction. Our model is simple, but describes a very complex, multi-stable energy landscape, using sophisticated numerical optimisation methods that overcome the difficulties caused by instabilities in large systems.

[H) Significance]
Our work opens up new ways of understanding the unique biomechanics of
fibrous-network ECM, by fully accounting for nonlinearity and associated loss of stability in fiber networks. Our results provide new insights on tumour invasion and metastasis

---

Comment #8: The issue I raised in this feedback was more concerned with addressing the reproducibility of the work in general. Qualitative prediction is subjective, and if that's the best that can currently be done, then so be it. I am asking the authors to address the issue per se. Reproducibility (or the lack thereof) in biology is a rapidly growing concern, to which biological modeling is not immune.

We recognise the importance of this comment and the Reviewer's concern on reproducibility.

While our previous work [Grekas et al.] included experiments through collaboration with an experimentally capable team that performed them (Notbohm-Proestaki), the present work comprises only theoretical modelling and numerical simulations, as our collaboration with the experimental team has been inactive for some time. Our core group consists of applied mathematicians and computational scientists (engineers and applied mathematicians) but no experimentalists.

Quantitative predictions. Our model predictions do include quantitative statements that are compared to available experimental data. An important example is the prediction of the cell contraction level required to produce patterns such as tethers, as well as the densification ratio magnitude within densified zones. These show that the version of our model with unstable single-fiber response is closer to experimental observations than the version with stable fibers. Another model prediction we included in our first revision was the spatial decay rate of ECM displacements, which compares well with older experiments.

*Qualitative prediction is not always subjective. In mathematics (geometry), an important qualitative characteristic of a pattern that is well defined and objective is its topology. Pattern formation has topological features that are objectively identifiable, and here we have excellent agreement: split multiple radial "hairs" instead of a "*homogeneous halo*", and axially split tethers versus a solid band, observed in [Grekas et al.] are not predicted by most previous models, but are evident in our simulations.*

Also, trends in certain pattern changes and observable geometric quantities as parameters are changed; do they increase or decrease? Are there topological changes, such as hair refinement for larger particles (see Methods, Size Effect) or a continuous tether joining particles vs an incomplete road with gaps? Some of these questions have an objective qualitative answer. Here our results are in qualitative agreement with experiments. Sometimes the answer is quantitative; see Fig. 7e graph.

Minimal models versus "quantitatively informed" models: In order to make precise quantitative predictions, a model should preferably have quantitative experimental input for its parameters. Here the quantitative input would be experimental data on the stress-stretch relation of a single fiber, as well as their statistics due to variations between individual fibers in the ECM. While limited, such data are available for fiber tension, but virtually impossible to collect for compression, because of buckling instability at a microscopic scale, which renders experimental measurement extremely difficult, due to extremely low forces below the sensitivity of current measuring techniques. Our results, and those of many previous models, agree that fiber compression plays a central role in ECM mechanics due to buckling. It is thus currently next to impossible to have a substantially "quantitatively informed" model. The alternative is a qualitative model that captures trends in single-fiber behavior. Our model is precisely that: a minimal, qualitative model at the single-fiber level, that manages to predict general trends and topological features of observed patterns in a complex fiber network, but also seems to capture limited quantitative observations, as mentioned above. It is possible to incorporate additional effects, such as the energy cost of angle changes between crosslinked fibers, but there is apparently a total lack of experimental data for this. Given these uncertainties, we feel that our minimal, qualitative model captures more aspects of observed behaviour than previous work. Our model is actually a whole family of different specific sub-models (Families 1 and 2) and we learn a lot through comparison of different sub-family predictions.

How can someone else implement the model as described in the manuscript and assess whether they have done so correctly?

We are not sure what the Reviewer means by implementing the model. Are more details about the mechanics model or its computational implementation desired?
We have included our numerical code and straightforward, detailed instructions on how to input relevant data, such as mesh size (fiber length), contracting particle diameters and distances and contraction levels in GitHub. We have also included all such data used in all simulations presented in the paper.

How could predictions from the model be quantitatively compared to experimental results? If the answer to such questions is "they can't", then why not? Is there no well-defined technique using image analysis, morphometrics, etc., to address these limitations?

At this moment, important data required for further quantitative comparisons (see <u>Quantitative predictions.</u> above) is not available. For example, fiber density vs light intensity from experimental images would be required, and this was not obtainable by the experimental group. There are, however, techniques that could be used in new experiments. We would need new experimental measurements of spatial variation of ECM displacements using optical techniques that measure displacements, (such as Digital Volume Correlation, see Notbohm PhD thesis 2013 for a description). This cannot be extracted from available data. We would also require detailed information on collagen network properties, such as fiber statistics on length and thickness, and network connectivity among others. These varied from specimen to specimen in experiments presented in Grekas et al. Also new simulations <u>designed specifically</u> to describe those experiments would need to be performed. We would also need a three-dimensional network model and 3D simulations (very demanding computationally, but currently in progress by our group) to compare to experiments using contracting active spherical particles in a 3D matrix, where it would be difficult to extract detailed quantitative data from the ECM interior as opposed to the surface of the ECM. Also, as discussed above, post-buckling behavior of individual fibers would be required in principle, though this is currently beyond experimental capabilities. We have included new references with experiments on single-collagen fibril buckling that we feel support our model's assumptions (papers suggested by Reviewer 3). See Methods, Evidence for Family-2 Unstable Fiber Buckling Model

==f
Reviewer #2: The review is uploaded as an attachment.

The authors have done a good job in revising their paper. I especially appreciate the more in-depth explanation on the stability of buckling.

We thank the Reviewer for the request to better explain (in)stability of buckling as it has led to improvement of the paper.

However there is still one major point I would like to have clarified: the interpretation of previous experimental work and how it relates to the model.
1) To make sure we are on the same page, I have included a sketch:
In this sketch, two contractile particles (cells or artificial particles; black) are connected via a densified tether (green). In addition, following the terminology suggested by Grekas et al 2021, the thin densified bands around the contractile particle are "radial hairs" (blue).

I fully agree with the authors that a densified tether is seen in experiments, both with single cells, multicellular aggregates, and artificial contractile partiles as used by Grekas et al 2021.
However, I disagree on the extent of radial hairs in experimental data, particularly at the length scale of cells.
<u>In order to rule out such causes as migration or filopodial extension, we used artificial contracting particles in Grekas et al 2021, and found heterogeneous densification patterns as well, see Fig 4a, 4d, 5b, 6b in that paper, where hair-like microstructures emerge around contracting particles.</u>
Closely looking at the figures that the authors mentioned (Fig 4a, 4d, 5b, 6b in Grekas et al 2021) I see a <u>homogeneous "halo"</u> of radial densification around the circumference of the artificial contracting particle that does not spread very far into the ECM, at most up to 1 diameter, while in simulations the radial hairs are inhomogenously distributed along the circumference of the particle, and spread quite far, clearly at least two diameters, into the ECM (e.g. comparing Figure 5b and c in Grekas et al 2021).

1)To be honest, we agree with this comment only regarding Fig.4a in Grekas et al., but <u>we do see radial lines emanating from particles</u> in Figs 4d, 5b, 6b, ibid. They are faint and rough, but they are quite different from the random blobs of green far away from particles and between particles above and below the tether. It helps to view images from a distance. We also recall that resolution of photos in the published version of Grekas et al. was unsatisfactory, and we complained to journal production to no avail. Inspection of Fig 1a, 1c ibid. reveals long hairs. We show clearer versions below.

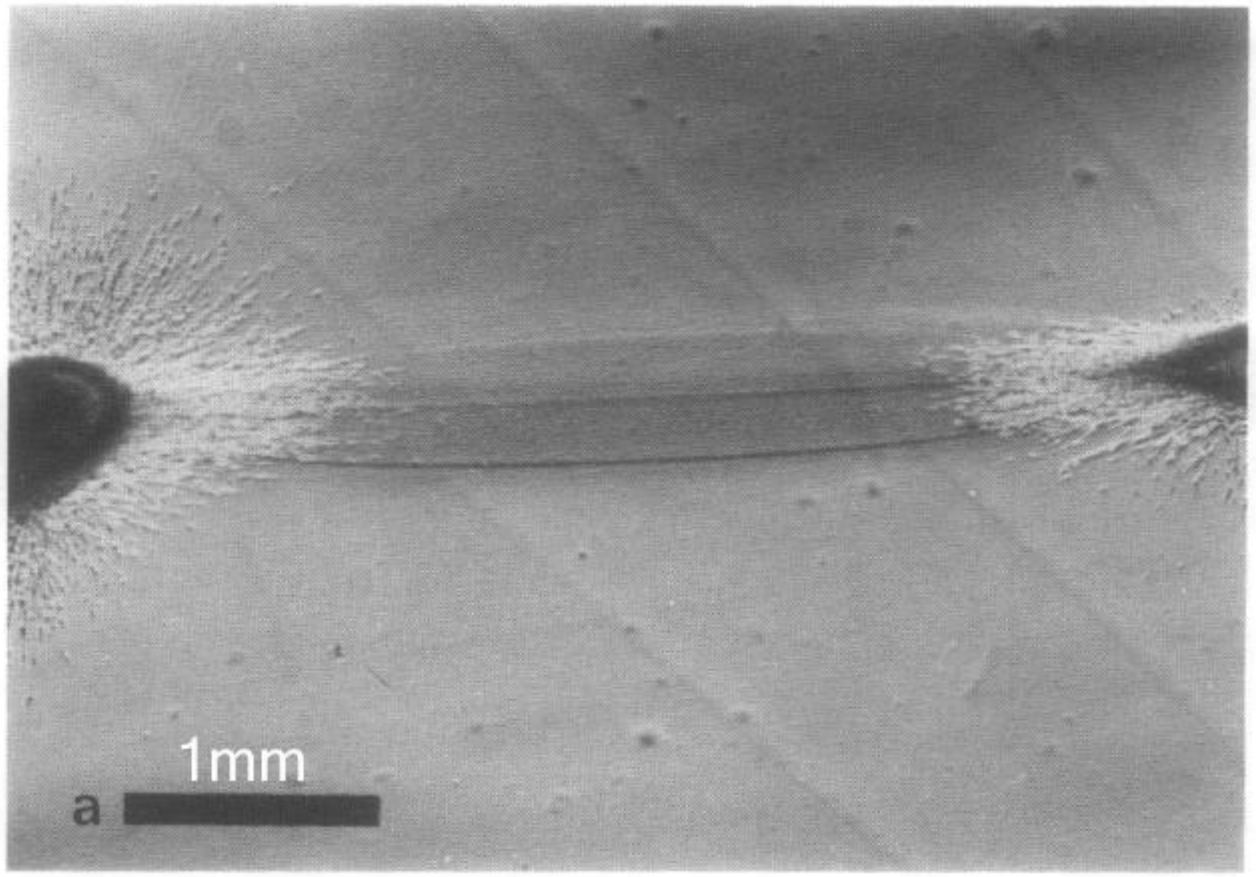

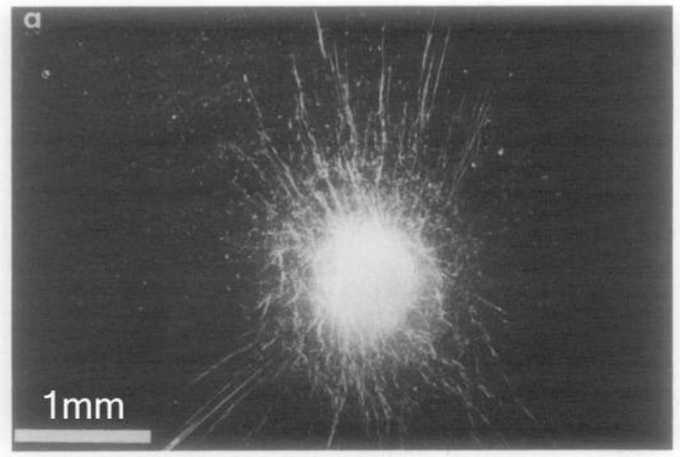

Radial hairs are evident as thick and thin radial lines emanating from particles in Fig.4d below. If you look in the middle between particles but above and below the tether, you see random blobs of green on a black background, whereas there are definite inhomogeneous radial structures emanating from each particles.

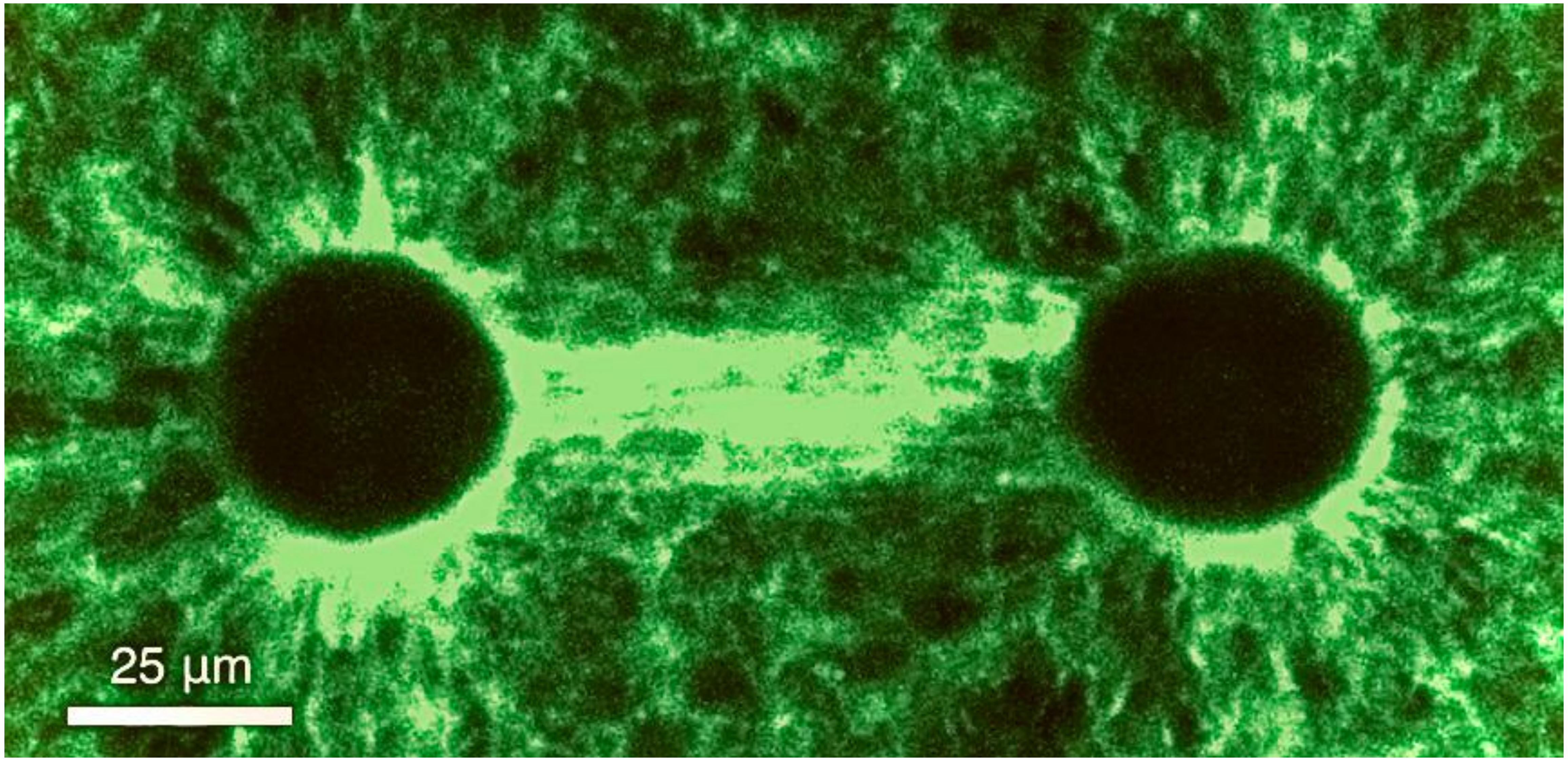

Perhaps less so in 5b, 6b but definitely hairs are there, although less evident.
Here is an unpublished example with evident radial hairs: (Proestaki, private communication).

Below:Unpublished (Courtesy M.Proestaki, private communication). Radial lines are quite evident

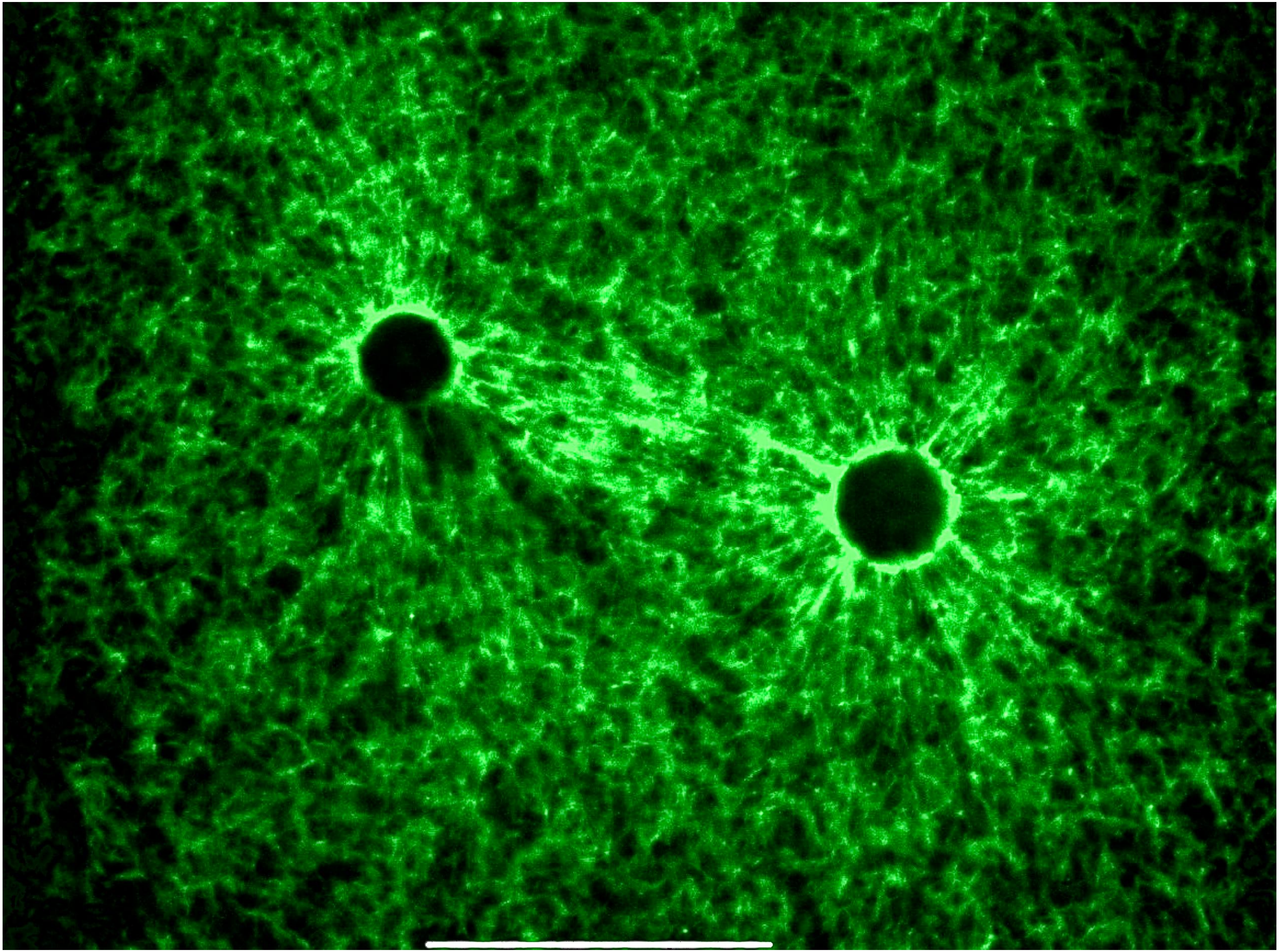

2) Radial pattern inhomogeneity. There is another reason why radial hairs appear much more inhomogeneous in simulations. This is because in experimental images, it is collagen fibers that appear light, whereas in simulations, we plot area densification ratio, which can be high even when fiber density is relatively low. We do this because area densification ratio is an indicator of both fiber collapse and fiber element collapse occurring in our model networks. Below we show two new attempts to depict fiber density from our simulations (right) compared to Fig 5b (below left, experiment with active particle) of Grekas et al. Unfortunately, the light intensity versus fiber density curve from experiments was not available, and there is a subjective choice of brightness scale in both photos and simulations. However, it is clear that there are radial hair structures in both. Their apparent length depends on brightness vs fiber density scales that are to some extent arbitrary, but the topology and pattern is similar and much more objective. Below left, we have increased the experimental image contrast to emphasise the non uniform fibers emanating from the particle. Here the simulated fiber density (right) is quite similar, but closer to a homogeneous halo than the area densification pattern in the figure after this one.

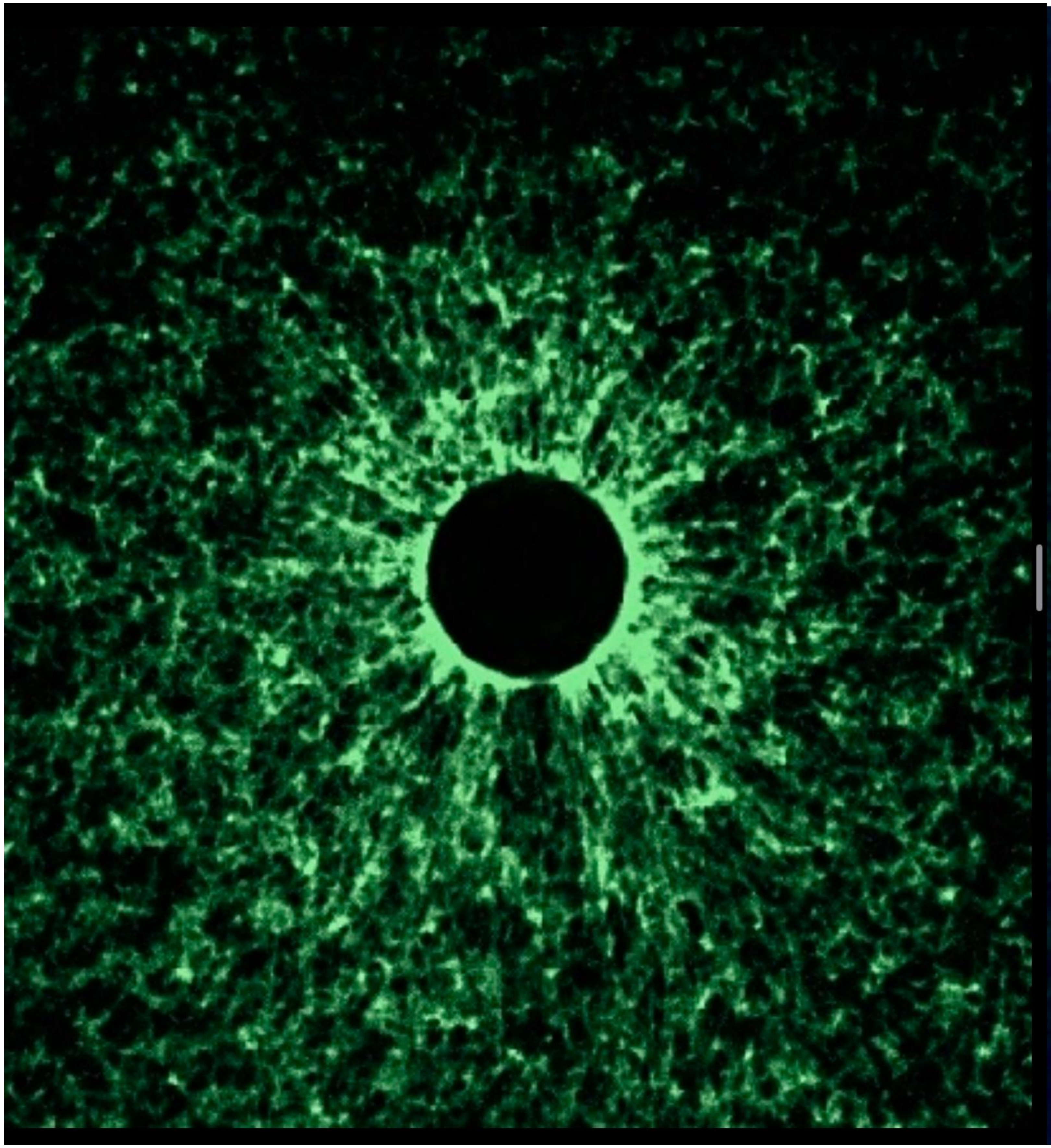
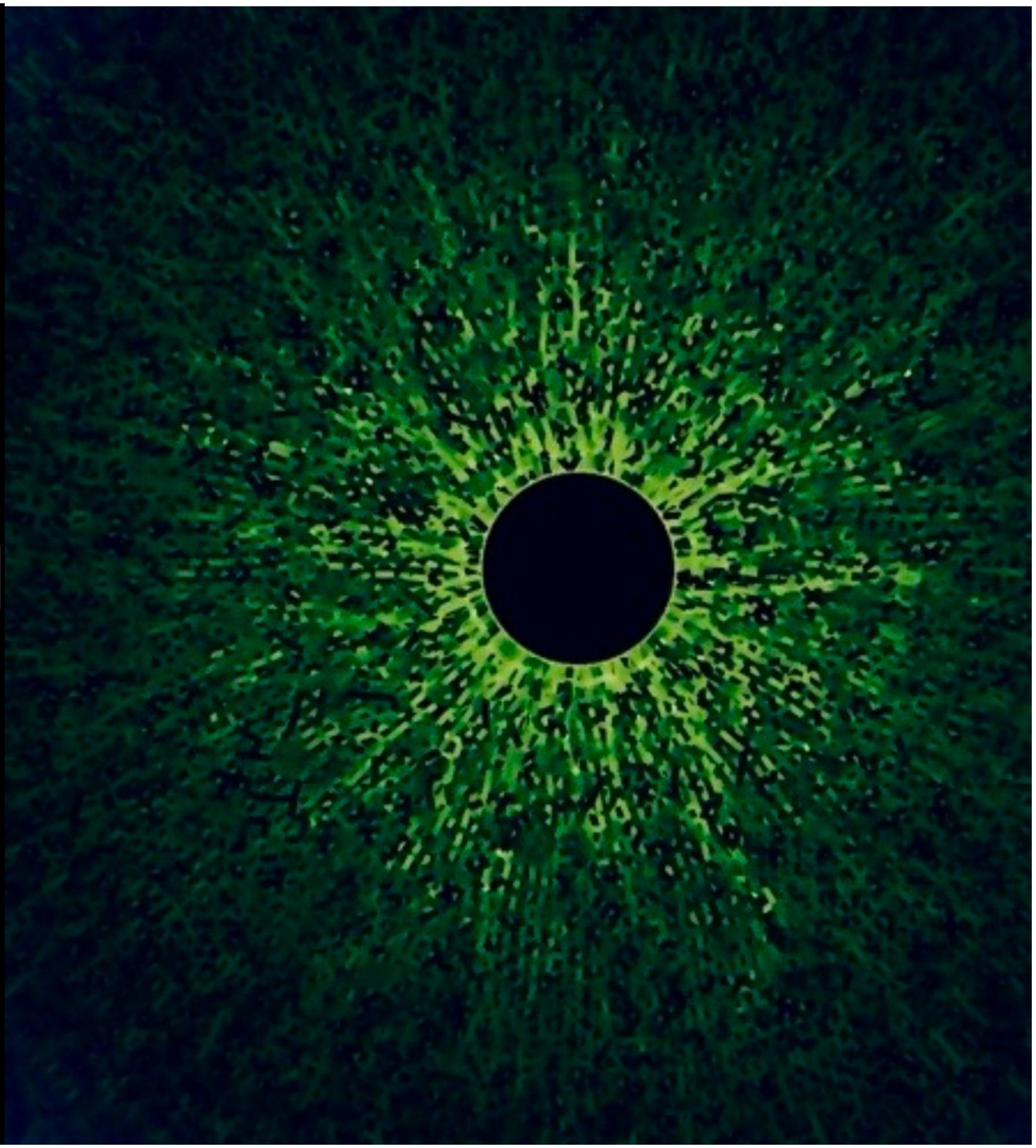

Below we show area densification (left) and fiber density (right) from the same simulation. It is evident that area densification ratio is much more inhomogeneous than fiber density, and densified tracts appear fewer and further apart. However the length of densified tracts as defined by area densification is much more objective (independent of brightness scale and photographic scales such as exposure, contrast, brightness etc). Also it is more difficult to compare it to available experimental data, which are sensitive to photography and its electronic reproduction at various stages, while the light intensity vs fiber density scale was never obtained.

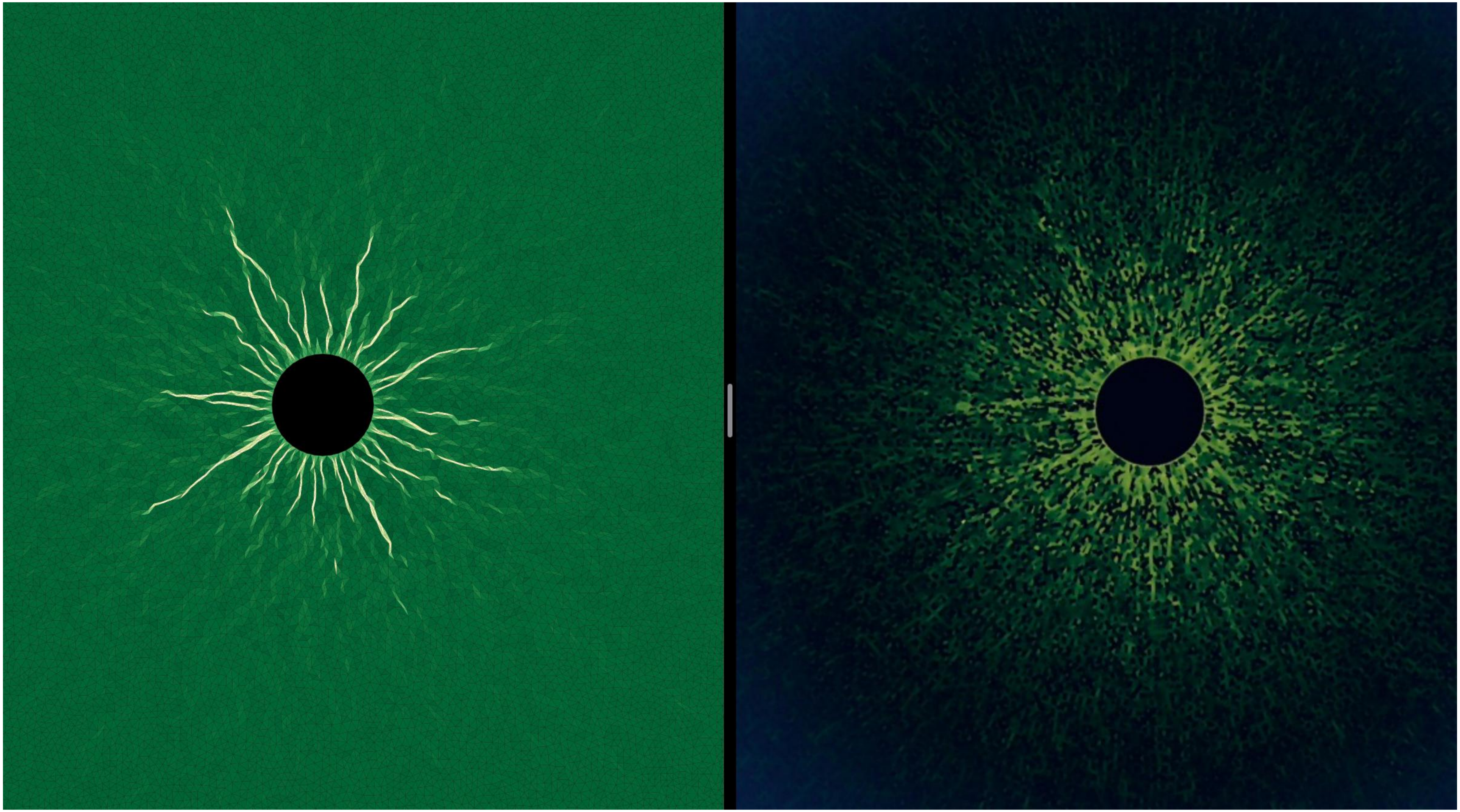

Below is another example with the original experimental figure (5b, unaltered) but larger simulation mesh scale (longer numerical network fibers). Average fiber lengths varied from sample to sample in experiments in Grekas et al. Brightness scales were chosen differently in these two examples (above and here). Here the experimental image (right) is not altered.

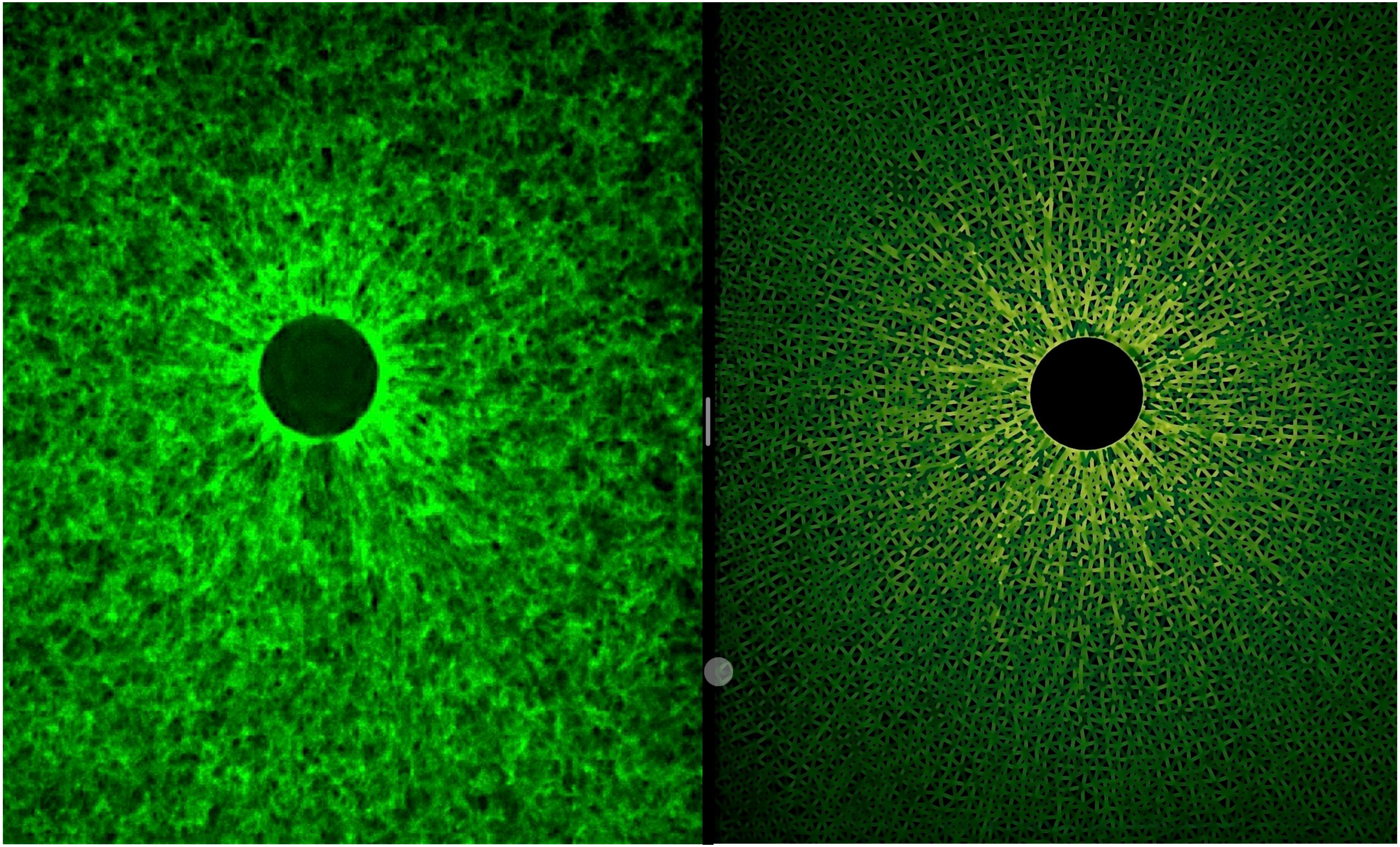

Again, densification ratio and fiber density from a single simulation, same as above

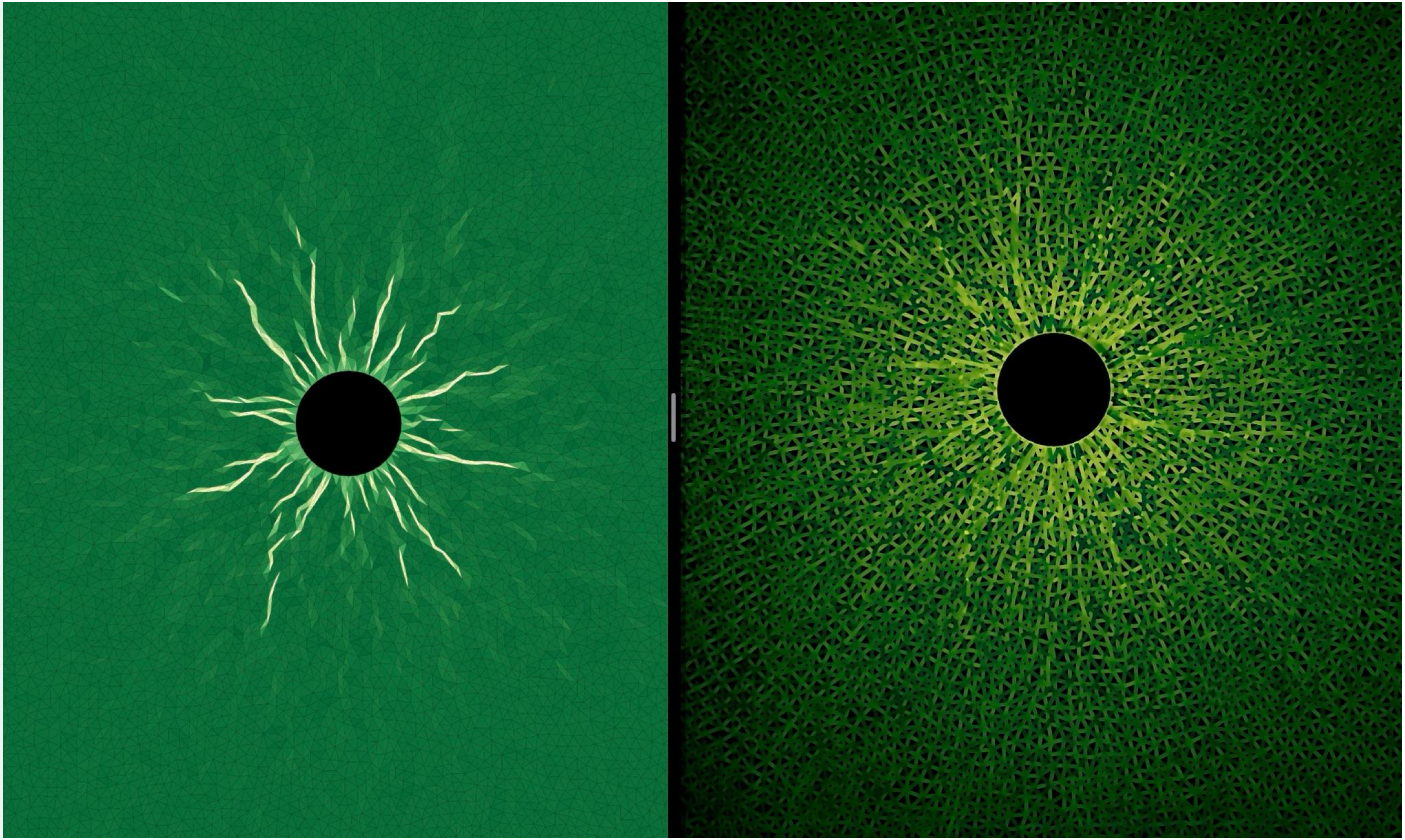

<u>Sub-model dependent hair length.</u> *The length of hairs in simulations depends on the sub-models used, even within each Family. See Supplemental Fig. S4 where at 50% contraction (same fiber and particle scales and 50% contraction the hairs due to the λ^7-λ^5 Family 2 model are twice as long as those of the λ^5-λ^3 Family 2 model, whereas all Family-1 models exhibit very weak densification. Below from Fig. S4, left 5-3 model. right, 7-5 model, both at 50% contraction, same network. Both are family 2 models, the 7-5 has hairs that are twice as long, and the 5-3 is closer to experimental observations.*

5-3 Model. *7-5 Model*

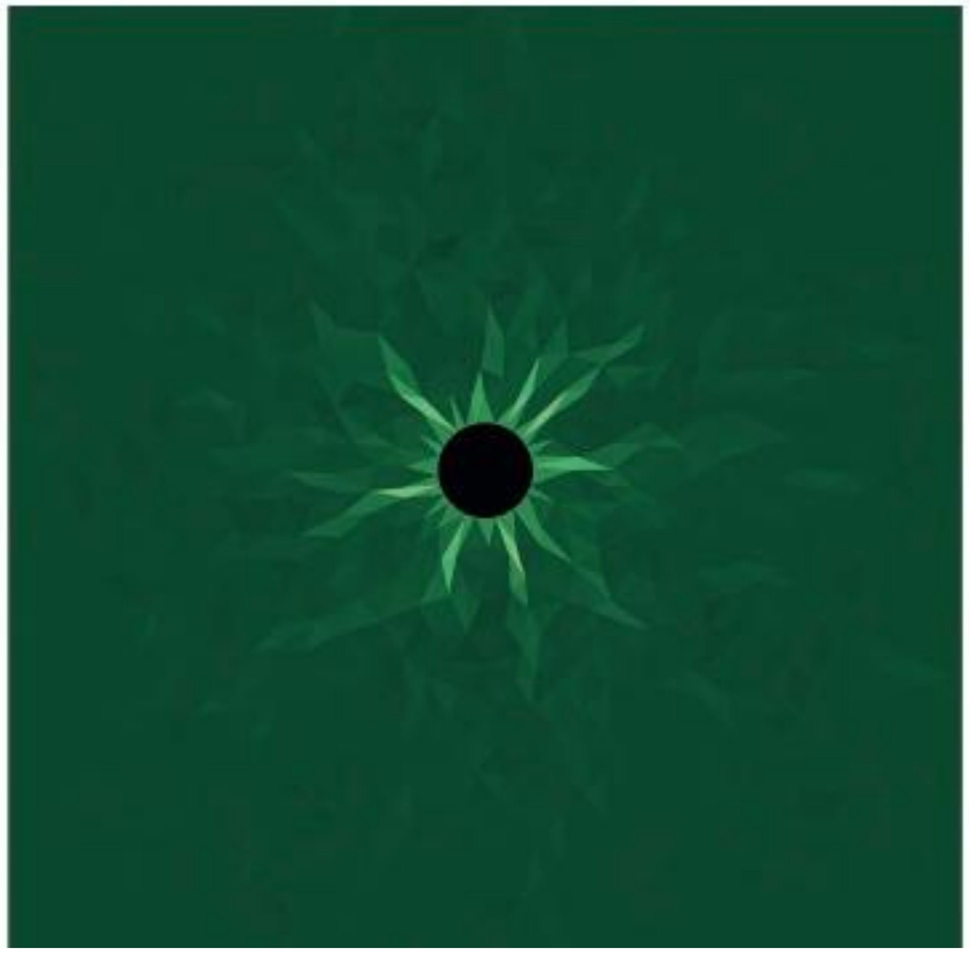
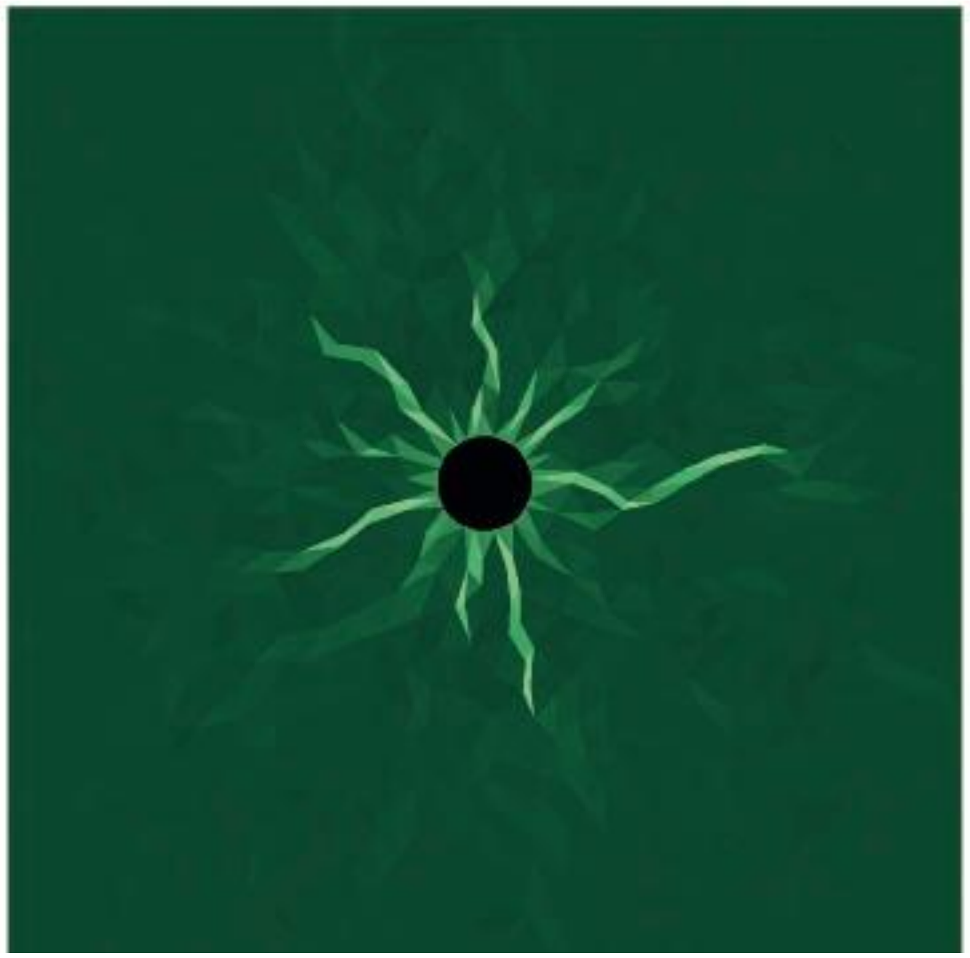

We have widely used the 7-5 model for many figures in the previous revision, but it is true that the 5-3 model (also Family 2) is often much closer to experiments. We have shown results with the 5-3 Family 2 model which are closer to experiments, in modified Figs 3, 6.
See also Fig. 7e which shows that continuous tethers are formed at observed contraction levels around 50% at a distance of about 8-10 cell radii with Family 2 models, compared with Family 1 which require unphysically high contraction levels above 80% to produce continuous tethers at that distance between cells. See graph in Fig 7e.

Compared to these experimental results, the data of family 1 models in the submitted manuscript, particularly Figure 3a-b visually resemble the experimental data in Grekas et al 2021 the most. Very long radial hairs, as displayed by family 2 models (e.g. Figure 3c), do not seem to occur at the cell scale.
Furthermore, this is also clearly explained in Grekas et al 2021 as a scale effect. The scale of multicellular aggregates is larger than the scale of single cells and the width of the thin hairlike bands emanating from them, hence multiple hairlike bands are observed emanating from larger clusters or artificial robotic cells (particles) Figs 1c,4a,4d,5b,6b in Grekas et al.
My argument is that the pattern of radial ECM densification in experiments at the scale of single cells resembles the pattern of family 1 models. The very long and inhomogeneous radial hairs of family 2 models do not resemble the data at the cell scale.

As noted above, Family-2 model 5-3 has much shorter hairs than its sibling, Model 7-5. More importantly, because of multiple length scales, the comment on cell-scale vs larger particle scale hairs is due to a size/scale effect.

Scale Effect. ***See new Size Effect subsection, lines. 367-387, and Fig S10. There are al least two characteristic lengths in this problem. Fiber length (characteristic network size) and particle diameter (individual cells vs multicellular clusters such as mammary acini (Shi et al.) , or artificial contracting particles (Grekas et al.) in the case of a single particle. For a particle pair there is also distance between particles (Graph in our Fig 7e). In simulations there is the additional length scale of computational domain size. The ratio of fiber to particle size has a major effect on the extent of densification patterns. Here we show that small enough particles produce very limited densification with few hairs (Fig. S10a below, with particle size a few times larger than fiber size, just two densified protrusions with densification ratio above 2.4. This is reminiscent of single cell behaviour. At the same contraction level, much larger-scale particles compared to fiber size (100 times larger) produce many extended fine hairs. See subfig b below for the same 5-3 model and c for the 7-5 model with equally fine, but substantially longer hairs (both are family 2).***

Suppl Fig. S10

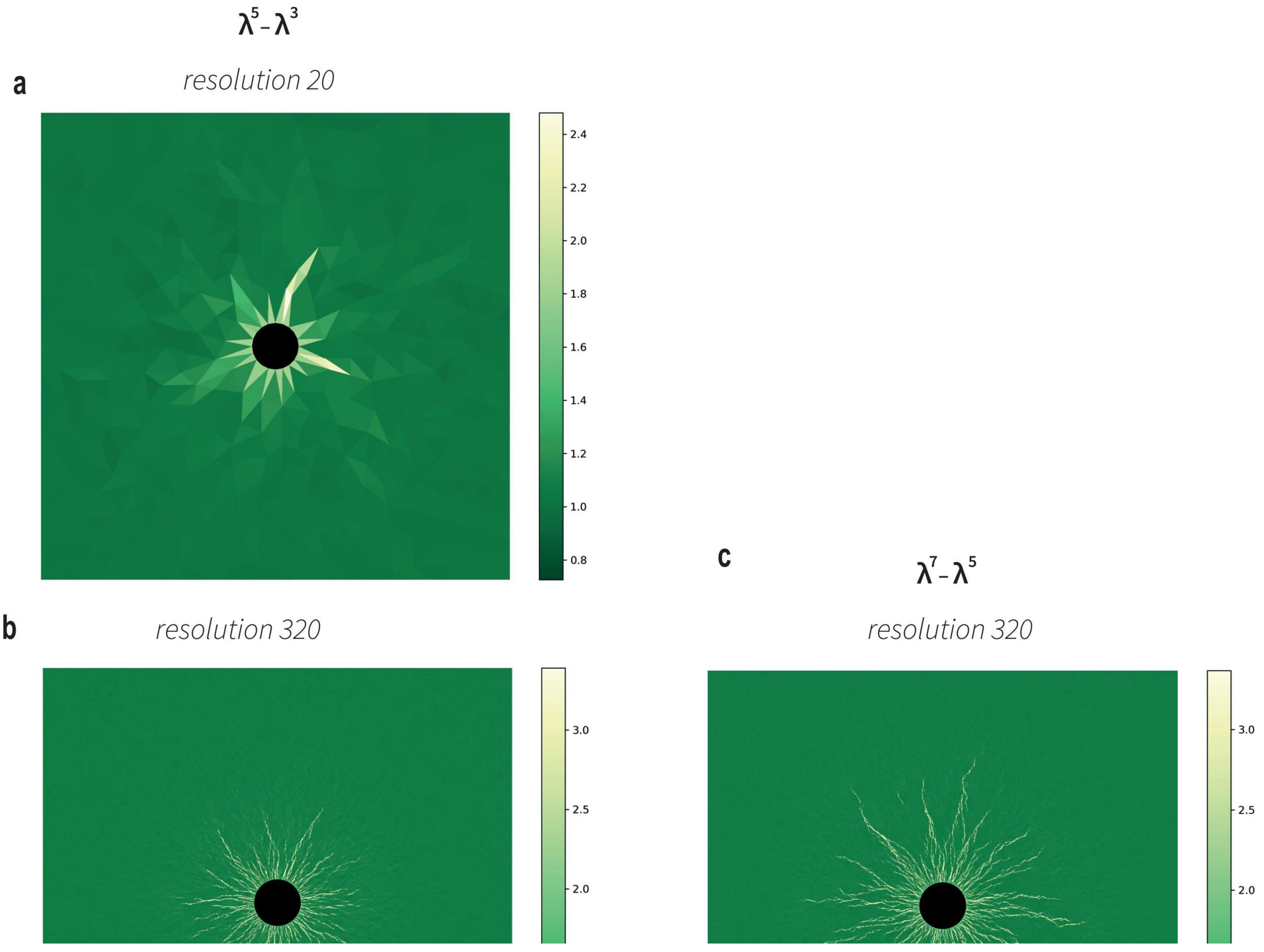
λ5-λ3
a
resolution 20
2.4
2.2
2.0
1.8
1.6
1.4
1.2
1.0
0.8
b
resolution 320
3.0
2.5
2.0
c
λ7-λ5
resolution 320
3.0
2.5
2.0

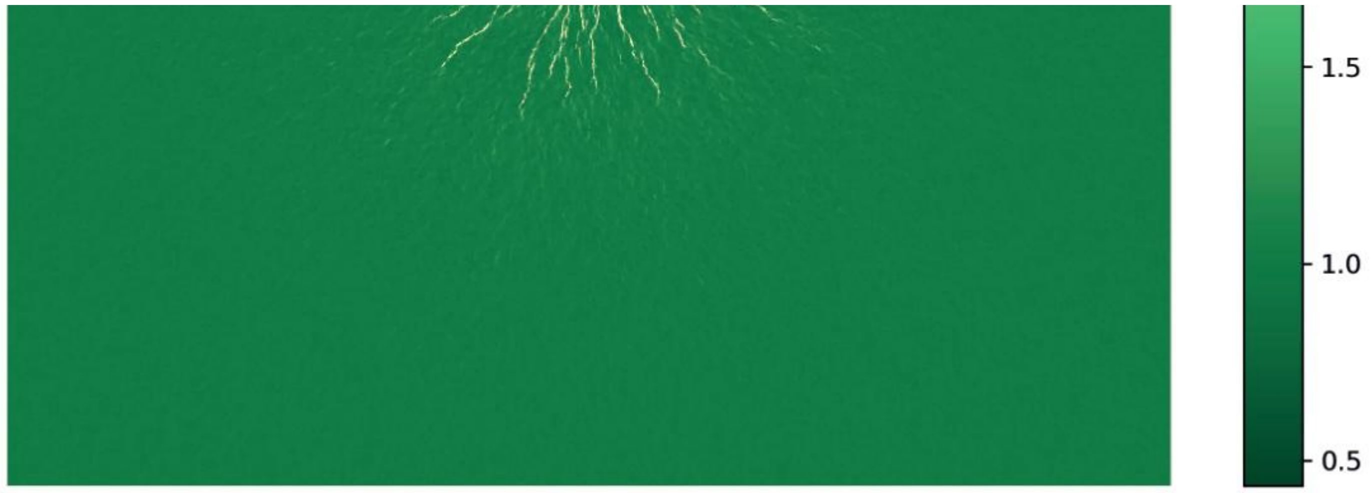

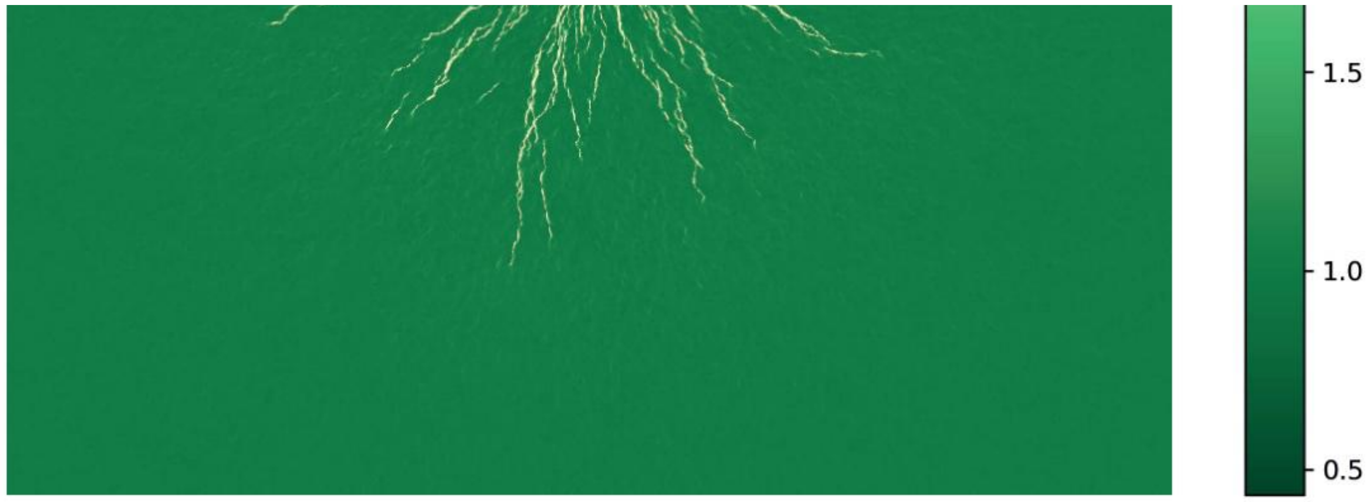

The fiber density pattern for the large particle in b above is closer to a homogeneous halo as e.g., in Fig. 4a in Grekas et al (largest particle imaged). This is because radial hairs are numerous and very thin compared to particle size that they cannot be discerned at the large scale.

In contrast in Notbohm et al. 2015 in experiments with isolated fibroblasts, a single densified tract joining adjacent cells appears immediately upon cell contraction, and hours afterwards filopodia extend slowly along the densified tract; Fig. 5 Notbohm et al. 2015.
The cited experimental work in Notbohm et al 2015 shows an earliest timepoint at 5 hours, not immediately upon cell contraction, and the GFP marker shows filopodia already at this timepoint that clearly overlap with the location of the radial hairs (and also along the tether). From these data it cannot be excluded that the radial hairs in this experiment formed due to filopodial activity.
This should be corrected in the discussion in line 669-671.

"Hours afterwards" has been modified to "subsequently" according to the original statement in Notbohm et al. for reasons explained below:

The statement is based on visual observations by microscopy. While no photos were taken before 5 hours showing the tether without filopodia, it was observed and stated in Notbohm et al that first a tether forms and subsequently filopodia (protrusions) grow along it: We quote from notbohm2015: " enables cells to induce … linear, tether-like paths that lead to other cells. … In our experiments, we have observed pairs of cells forming pointed protrusions along these tethers and subsequently growing toward one another…. The tether formation mechanism… results in greater tension and fibre density that is highly polarized in the direction of neighbouring cells (figure 5). Both tension and fibre density may provide a directional signal: by growing protrusions along the direction of the tethers, cells have a higher chance of approaching one another. The fact that cells change shape and grow along such tethers supports the hypothesis that they use this very same mechanism to sense and even approach their neighbour…"

There is also ample evidence from contracting artificial particles which cause tethers to form between them as shown in Grekas et al (and agreed by Reviewer 2: "I fully agree with the authors that a densified tether is seen in experiments…with… artificial contractile partiles as used by Grekas et al 2021"***) without the possibility of filopodia. Hence filopodia are not necessary for tether formation in fibrous collagen ECM.***

Shi et al. (doi:10.1073/pnas.1311312110) ***in a supplemental movie show tethers forming simultaneously with multicellular acinus contraction, followed by cells from it migrating along the tether afterwards.***

------------------------- Minor comments:
1) Line 57: What does "the latter" refer to?
2) The paragraph from lines 59-80 would benefit from clarification that the mentioned "fibers" are not biopolymers, since the references are from the engineering and mechanics literature. For example, the concluding statement (lines 76-80, "These studies strongly suggest that the mechanics of single collagen fibers…") can be misread to imply that measurements were done on collagen fibers.

We have removed "strongly", but added evidence from collagen fibril buckling experiments: Reviewer 3 brought to our attention experimental studies of collagen fibril buckling that we feel support our unstable buckling hypothesis. We also include analytical models that predict this observed behavior for unstable beams.

Please see red text in Methods, subsection Evidence for Family 2 Unstable Fiber Buckling Model.
and also in the Introduction.

Also, please see additional evidence in response to Reviewer 3 below, including experimental results on collagen fibrils and analytical results predicting them, supporting our hypothesis. This evidence with references has been discussed in the revised paper: Methods, subsection <u>Evidence for Family 2 Unstable Fiber Buckling Model.</u>

3) Line 101: What is "buckle collapse"?

Corrected to "buckle and collapse"

4) Line 177 is the first mention that stiffness is equivalent to the slope of the force-stretch relation. I suggest to mention this earlier, e.g. after Equation 2 is introduced, to make it easier to understand for readers from a biology background.

Done

5) Line 421: "not" should be "note"

It should be "not " (as in "not observed") but we rephrased it to make it clearer

6) Line 500: "Most ECM fibrous networks have connectivity that is between 3 and 4" requires a citation.

Added Jansen et al. 2018.

7) Line 505: "S9 Fig." should be "Fig. S9" 8) Line 611: Remove "refs"

Done

9) Line 642: "but resulting densification" → " but the resulting densification"

Done

10) Lines 642-643: "much sharper delineated" → "much more sharply delineated"

Done

11) Line 675: "philopodia" → "filopodia"

Done

12) Line 678: "displacments" – "displacements"

Done

13) Line 679: What does "these works" refer to?

Removed (vague)

14) Line 680: "philopodia" → "filopodia"

Done

===

Reviewer #3: In my previous review, I showed a concern about lack of evidence for the snap-through collapse of ECM fibers and the dependence of the ECM model on the unverified assumption.

We appreciate the Reviewer's concern and will attempt to address it. Our model comprises two sub-models. Family 1 with stable fiber response, and Family 2 with unstable fiber response, considered unverified by Reviewer 3. Our idea is to compare the two models, in order to see which one better agrees with observations. We found that the Family-2 model is superior in predicting experimental observations. We also identified a new instability mode, at scales larger than that of single fibers, which may occur even if single fibers are stable, and may play a role in ECM remodelling. Our theme is that instability mechanisms (which have been largely ignored in past models) are likely to be very important in fibrous ECM.

Reviewer 3 kindly provided two very interesting references (see below) which experimentally investigate collagen fibril buckling on a contracting substrate. Reviewer 3 states below that the results are consistent with buckling of linear beams.

However, as we show below:
1)This assertion (linear behaviour) is contradicted by statements in one of these studies and results in both.

2)We explain in detail why we find considerable evidence in these papers in support of the unstable model of fiber buckling (viewed as unverified by Reviewer 3)

In their responses, they argued that an assumption without direct evidence can be meaningful if it enables the model to reproduce experimental observations, and that there is indeed indirect evidence for their assumption.

Well, I have to agree to disagree with them.

We agree on this point!

There have been a few studies showing the buckling of collagen fibers with the formation of waves or loops:
[A]DOI: 10.1016/j.actbio.2022.06.044
and
[B]10.1021/acsnano.0c03695. I don't think that the observation of these patterns during buckling supports the snap-through collapse of collagen fibers. They look like the buckling behavior of linear beam structures.

Actually, we feel that these very relevant papers (***henceforth referred to as [A] and [B]***) ***provide further evidence for our Family-2 model of single-fiber unstable buckling. Specifically:***

1)The analysis used in [A], [B] ***to infer fibril material properties is based on a linear model. The authors*** of [A] ***point out that the linearity assumption is violated for large compression. Quoting from [A]: "In fact, it is known that collagen fibrils show highly non-linear stress-strain relationships [21]".***

It is shown in [A] that the sinusoidal form (consistent with linear beams) often does not persist for larger compressions. We quote from[A]: "This can be seen, for example, in Fig. 3d, where the fibril shows a more pronounced sawtooth pattern" (with corners, see Fig 3d reproduced below).This is not consistent with linear beam behaviour. This contradicts the reviewer's statement that "They look like the buckling behavior of linear beam structures". See Fig 3d of [A] reproduced below.

[A], Fig.3d

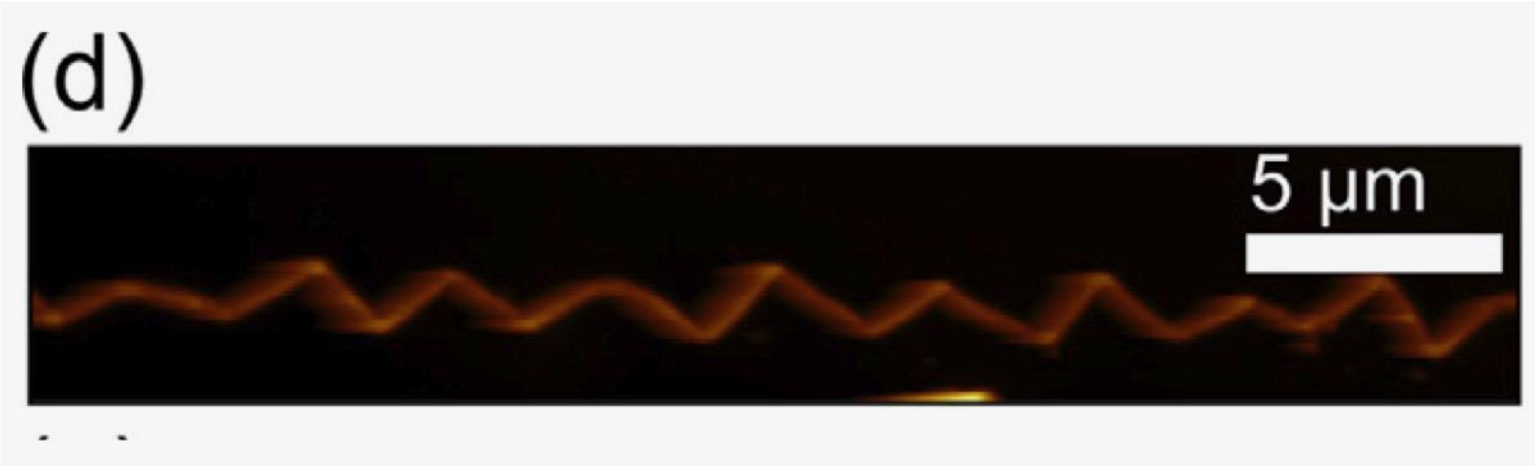

Also in[A], Figs 5c, 3e, evident deviations from a sine wave with curvature localisations are shown. These are consistent with the analytical model of Fosdick RL, James RD. The elastica and the problem

of the pure bending for a non-convex stored energy function. Journal of Elasticity. 1981 Apr;11:165-86, which assumes instability for a finite range of curvatures.

2)In [A] it is also emphasised that fibrils have a hierarchical structure. Here we observe that sharp curvature localizations and corners observed in [A] are also predicted by the hierarchical beam buckling study Tarantino and Danas , which we have cited as evidence of single fiber instability. They also predict a sawtooth shape; see below their Fig.10b.

Tarantino and Danas, Fig.10

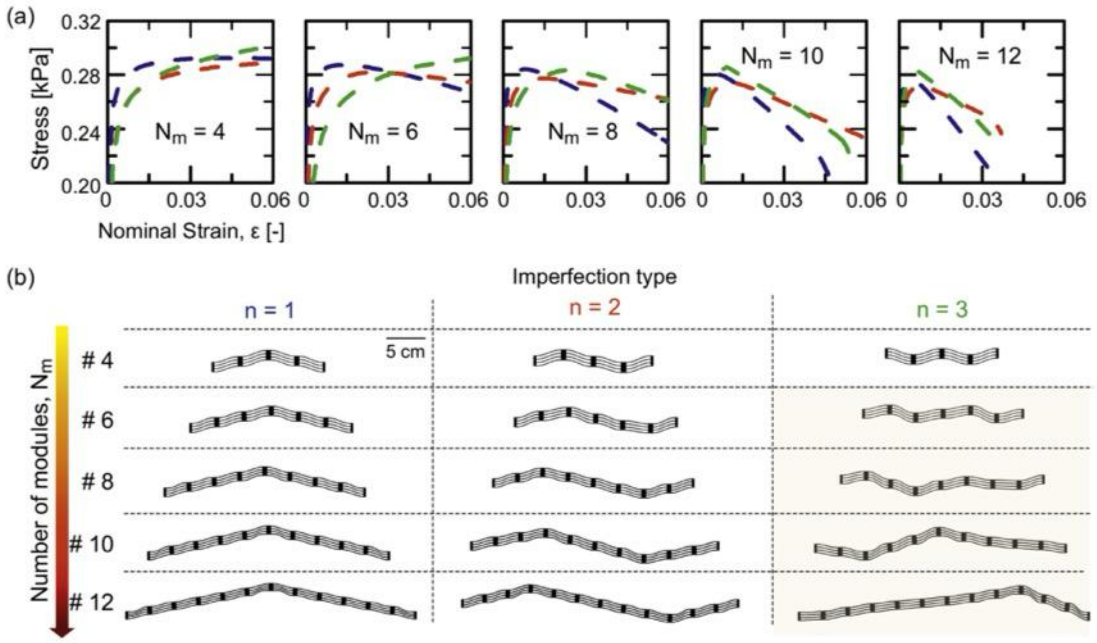

Fig. 10. Numerical post-bifurcation response of various hierarchical beams comprised of elemental beams with slenderness $\lambda = 50$ and $N_m = 4, 6, 8, 10, 12$. (a) Nominal stress-strain response (the colors blue, red and green correspond to imperfection-type $n = 1$, 2 and 3 respectively) and (b) post-bifurcation patterns at 0.03 nominal strain. The highlighted yellow area comprises all hierarchical systems whose deformation patterns do not correspond to the initial eigenmode-like imperfection. (For interpretation of the references to colour in this figure legend, the reader is referred to the web version of this article.)

3)In Fig.10a above, Tarantino and Danas find that the stress vs effective strain has negative slope for large compressive strain, which is precisely what we assume in our Family-2 model.

4)Sawtooth corners are a result of extreme curvature localization, which is characteristic of instability. This has been studied and predicted analytically by Royer-Carfagni:
[C]: https://doi.org/10.1016/S0997-7538(00)01128-1
It is predicted in [C] that an unstable beam with decreasing (unstable) moment-curvature relation exhibits corners (sawtooth) in its buckled shape. Such a beam can easily be shown to have an unstable stress-stretch curve in compression with negative slope as in Family-2 models in our paper.

Another simple example of unstable buckling involves a drinking straw, which collapses suddenly and forms a corner, where the inhomogeneous hollow cross section (a circle) collapses (flattens) at the corner , and loses bending resistance and compressive stress resistance, as in our family-2 models. Such a phenomenon is not predicted by linear beam models, but is predicted by the hierarchical beam studies we cited, and is observed in the experiments in [A]. As a result, [A] provides further experimental evidence in support of our model of Family 2 fibers undergoing unstable buckling. Graphene nanotubes also behave similarly. In many of these cases the cross-section, which is inhomogeneous, collapses from a circle to a flat segment and the beam loses bending resistance as a result. This is associated with a decrease in stress sustainable by the beam/straw/fibril.

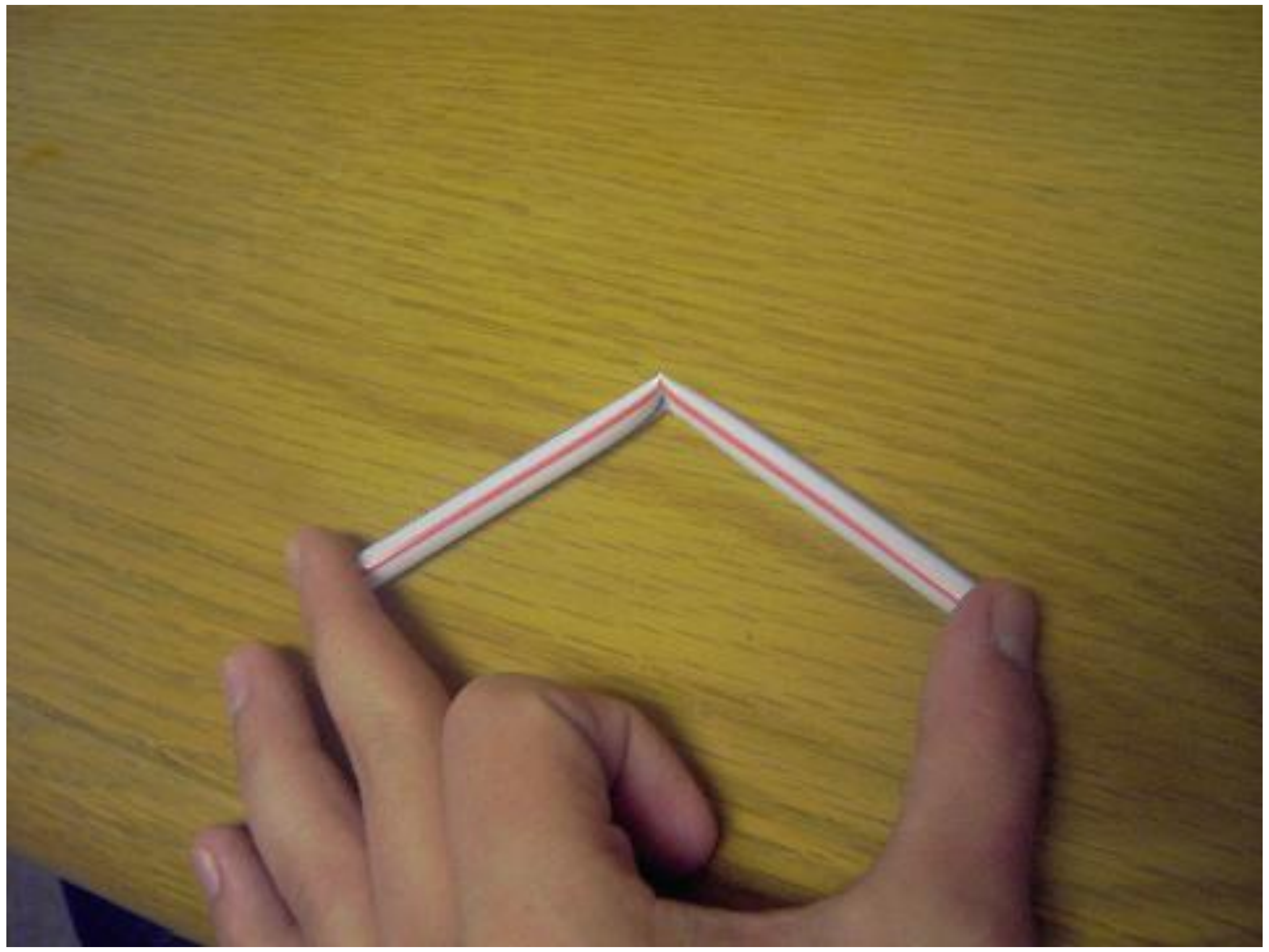

The indirect evidence that the authors referred to seems to be theoretical prediction or different structures. After seeing these experimental observations directly related to collagen fibers, it is hard to buy the authors' assumption even more.

Actually the evidence in [A] we just described is experimental, not a theoretical prediction. As explained above in points 1) through 4), in view of the findings of [A], Tarantino and Danas, and [C], it appears that our assumption of unstable fibers, in conjunction with simulations [Tarantino and Danas] and theoretical studies [C], is consistent with experimental observations [A,B], that are not consistent with linear/stable fiber models. In [B]: 10.1021/acsnano.0c03695 (cited by the Reviewer) similar behaviour with [A]—not consistent with linear behaviour—is presented, with fibrils forming multiple kinks upon compression.

Also in [B], it is stated that "In tendons, for example, collagen fibrils are packed into fibers and are linked to their neighbors by proteoglycans every 67 nm along their length." This is very reminiscent of the hierarchical beam structures studied in Tarantino and Danas, with multiple parallel "fibrils" reinforced periodically with transverse links; see above their Fig.10b for this structure. These exhibit unstable buckling as in our Family 2 models, and exhibit curvature localizations and sawtooth shapes (Fig.10b above), not predicted by linear models, but observed in experiments [A,B], and theoretically predicted by [C] for unstable beams. As a result, our Family 2 unstable model is suitable—as opposed to linear beam models—for predicting much of the observed behaviour in [A] and [B], supported by analytical models [C] and simulations [Tarantino and Danas].

The indirect evidence that the authors referred to seems to be theoretical prediction or different structures.

The "different structures" mentioned by the Reviewer include graphene nanotubes and ropes, both with structured (inhomogeneous vs solid) cross sections, and both mentioned in [A] and [B] and their references. So we see no reason to exclude them in drawing conclusions on the connection of inhomogeneous/hierarchical structure and instability. Here and in Tarantino and Danas, it becomes a common theme that the instability in buckling is closely related to the inhomogeneous/hierarchical structure. In all these cases, possibly including collagen fibers, the cross-section, which is inhomogeneous, collapses, and the beam loses bending resistance, and compressive load bearing capacity, as a result. We therefore find that, taken together, all the studies mentioned above provide considerable evidence, experimental and theoretical, for the validity of our unstable-fiber model.

If the assumption is not verified, I would consider a model based on the assumption to be a phenomenological model, which shows one of many possible ways to reproduce experimental observations at the ECM scale. Then, the impact of such a model would be limited.

The evidence presented above with aforementioned references added to our paper, has been included in the revised version. Please see the red text in the Introduction and also Methods, Evidence for Family 2 Unstable Fiber Buckling Model.

If one wishes to be objective and consistent, criticisms concerning lack of evidence certainly apply to the linear beam model mentioned by Reviewer 3. In fact, there is plenty of evidence against it, as it has long been established that fibers are highly nonlinear. At best, the venerable linear model is an outdated model based on tradition and mathematical simplicity, and possibly valid for <u>small deformations</u> of steel trusses and engineering structures, but it is becoming increasingly recognised that it is not experimentally justifiable in the regime of large deformations undergone by soft matter, such as biomaterials. However, many studies still use it for ECM networks. We feel that the time has come to go beyond it, to new hypotheses that account for the various instability mechanisms, some of which we identify in this work.

Have the authors made all data and (if applicable) computational code underlying the findings in their manuscript fully available?

Reviewer #1: **No:**

We have included our numerical code and straightforward, detailed instructions on how to input relevant data, such as mesh size (fiber length), contracting particle diameters, interparticle distances and particle contraction levels in GitHub. We have also included all such data used in all simulations presented in the paper.

Reviewer #2: Yes
Reviewer #3: Yes
